# A library of reporters of the global regulators of gene expression in *Escherichia coli*

**DOI:** 10.1128/msystems.00065-24

**Published:** 2024-04-30

**Authors:** Suchintak Dash, Rahul Jagadeesan, Ines S. C. Baptista, Vatsala Chauhan, Vinodh Kandavalli, Samuel M. D. Oliveira, Andre S. Ribeiro

**Affiliations:** 1 Faculty of Medicine and Health Technology, Tampere University, Tampere, Finland; 2 Department of Cell and Molecular Biology, Uppsala University, Uppsala, Sweden; 3 Joint School of Nanoscience and Nanoengineering, North Carolina A&T State University, Greensboro, North Carolina, USA; Danmarks Tekniske Universitet The Novo Nordisk Foundation Center for Biosustainability, Kgs. Lyngby, Lyngby-Taarbæk, Denmark

**Keywords:** global regulators of gene expression, transcriptional reporters, synthetic single-copy plasmids, native promoters, fluorescent proteins, strain library

## Abstract

The topology of the transcription factor network (TFN) of *Escherichia coli* is far from uniform, with 22 global regulator (GR) proteins controlling one-third of all genes. So far, their production rates cannot be tracked by comparable fluorescent proteins. We developed a library of fluorescent reporters for 16 GRs for this purpose. Each consists of a single-copy plasmid coding for green fluorescent protein (GFP) fused to the full-length copy of the native promoter. We tracked their activity in exponential and stationary growth, as well as under weak and strong stresses. We show that the reporters have high sensitivity and specificity to all stresses tested and detect single-cell variability in transcription rates. Given the influence of GRs on the TFN, we expect that the new library will contribute to dissecting global transcriptional stress-response programs of *E. coli*. Moreover, the library can be invaluable in bioindustrial applications that tune those programs to, instead of cell growth, favor productivity while reducing energy consumption.

**IMPORTANCE:**

Cells contain thousands of genes. Many genes are involved in the control of cellular activities. Some activities require a few hundred genes to run largely synchronous transcriptional programs. To achieve this, cells have evolved global regulator (GR) proteins that can influence hundreds of genes simultaneously. We have engineered a library of *Escherichia coli* strains to track the levels over time of these, phenotypically critical, GRs. Each strain has a single-copy plasmid coding for a fast-maturing green fluorescent protein whose transcription is controlled by a copy of the natural GR promoter. By allowing the tracking of GR levels, with sensitivity and specificity, this library should become of wide use in scientific research on bacterial gene expression (from molecular to synthetic biology) and, later, be used in applications in therapeutics and bioindustries.

## INTRODUCTION

For millions of years, bacteria have evolved complex transcriptional programs of stress responses. These programs are carried out by transcription factors (TFs). While most TFs regulate one to a couple of genes, less than 10% have evolved to directly influence tens to hundreds of genes (1). For example, in *Escherichia coli*, 22 TFs alone control 31% of all 4,747 genes (Table S1) (2). Hereafter, we refer to these 22 TFs as global regulator (GRs).

In general, GRs participate in complex, life-saving phenotypic changes (Table S2) (3, 4). For example, the GR “CRP” regulates hundreds of genes (2, 5, 6) influencing, among other things, how cells use carbon in the absence of glucose (7). This, in turn, enhances *E. coli*’s catabolic flexibility during slow, carbon-limited growth (8). Similarly, the GR “H-NS” influences approximately 200 genes (2) that particularly affect chromosome compaction (9) during osmotic, pH, and temperature shifts (10). To study how GRs influence the transcription factor network (TFN), one needs to track their levels over time.

Advances in synthetic biology (11–13) have led to the engineering of two large strain libraries for tracking the expression levels of many genes in live *E. coli* cells. One is the fluorescent transcriptional reporter library (“TR Library”) (14). It monitors the transcriptional activity of many natural promoters by having copies of these promoters (in low-copy reporter plasmids) controlling the production of RNA coding for fast-maturing GFP. The other is the “YFP fusion library” (15). It uses chromosomal integrated yellow fluorescent proteins (YFPs) followed by the native genes, both under the control of the natural promoters. Unfortunately, the first library only tracks 7 out of the 22 GRs mentioned above, while the second library only tracks 4 (out of which, 1 is also in the TR Library). Thus, they cannot be used to track most GRs using the same fluorescent protein.

The lack of probes (with the same properties) for all GRs is currently a major limitation in the study of the global transcriptional programs of the TFN of *E. coli* (e.g., for stress adaptation). Specifically, since GRs compose a large fraction of the TFN, tracking their levels over time is necessary to identify the triggers of phenotypic modifications that cells undergo during stress adaptation. For example, in reference (16), tracking σ^38^ levels overtime, using a fast-maturing fluorescent protein, allowed establishing that they only change after the beginning of the transition to stationary growth. Tracking more GRs would have allowed determining, e.g., if some of them changed prior to this transition (and, thus, potentially acted as triggers).

To address this, we aimed to engineer a strain library of fluorescent reporters to measure the single-cell transcription dynamics of the 22 GRs in *E. coli* controlling, arguably, more than 30% of all genes of *E. coli* (2). Of these, we were only able to engineer 16 functional GR reporters. These 16 arguably control 29% of all genes. The remaining six were non-functional (for reasons discussed below) and, thus, were not included in the library. The functional and non-functional constructs are listed in Table S1.

Each engineered strain contains a single-copy plasmid harboring a copy of the native promoter of the GR gene, including the operator sites, to incorporate the regulatory machinery of the native gene. This promoter region, unique to each strain, controls the transcription of a sequence coding for a fast-maturing green fluorescent protein (GFP) variant (17), common to all strains.

**Fig 1:**
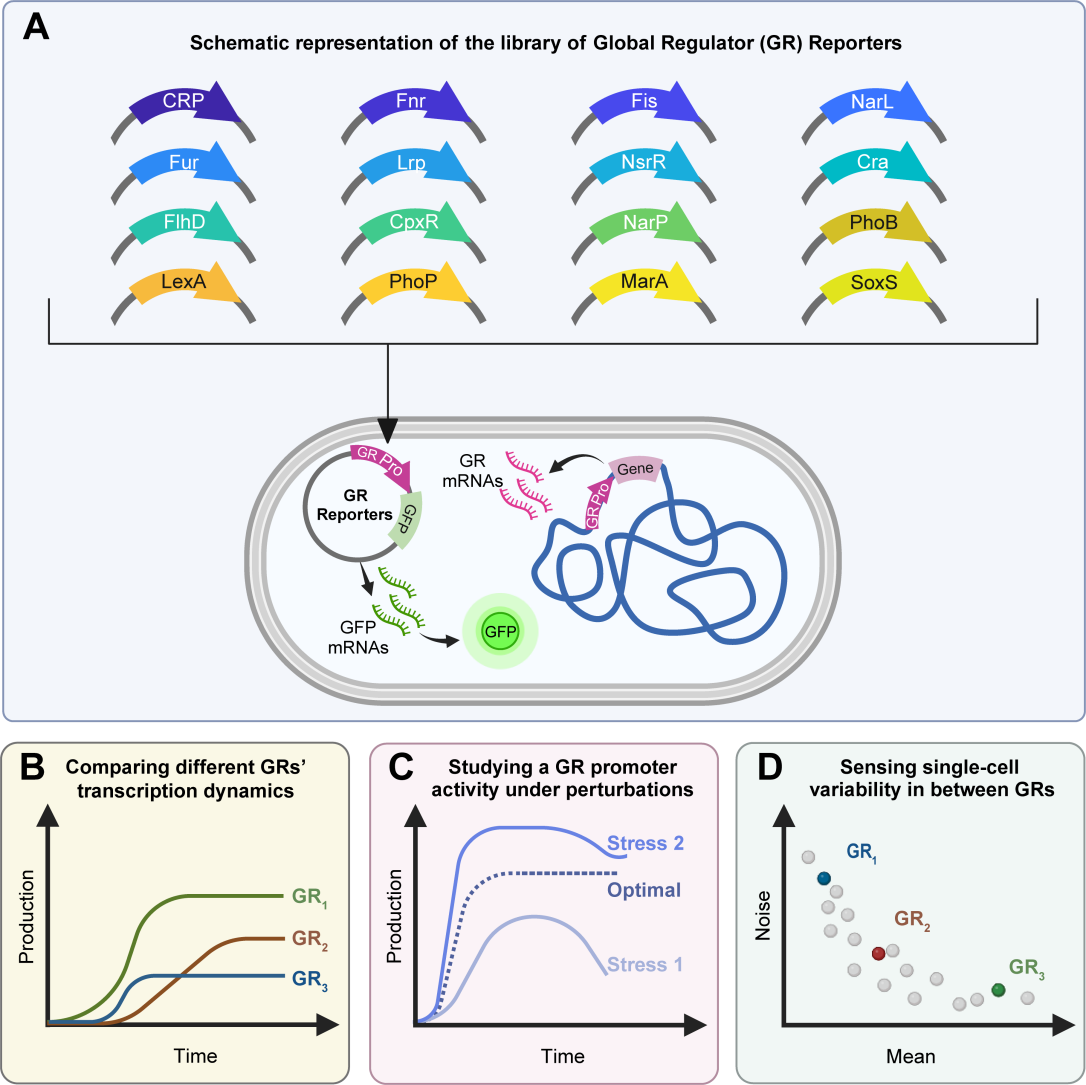
Schematic representation of the probing system supporting the library of GR reporters, along with direct applications of the library. (**A**) Each strain carries a single-copy plasmid that codes for the native promoter region of a GR. It follows the GFP coding region. The plasmids do not carry the coding region for the GR protein. As such, our constructs act only as reporters. From the average time-lapse fluorescence of cell populations from the different strains, (**B**) the library allows comparing the levels of different GRs over time, (**C**) the levels of a GR in different conditions, and (**D**) the cell-to-cell variability in GR levels. The figure was created with the assistance of BioRender.com.

Figure 1 informs on the 16 functional reporters and illustrates their sensing and reporting mechanism based on their promoter and production of fluorescent proteins. Also shown are potential applications and illustrative data for those purposes. Next, we describe how the library was assembled, and show evidence of its functionality in standard growth (commonly referred to as optimal) and stressful conditions. Figure S1 shows a flowchart of the steps of this study.

## RESULTS

### Assembly and testing of the library of GR reporters

We first searched in RegulonDB (2) for the number of genes known to be regulated by each TF in *E. coli*. Next, as mentioned, we defined as GRs the 22 TFs with 40 or more target genes each (in total, they control more than 30% of the genes of *E. coli*).

**Fig 2:**
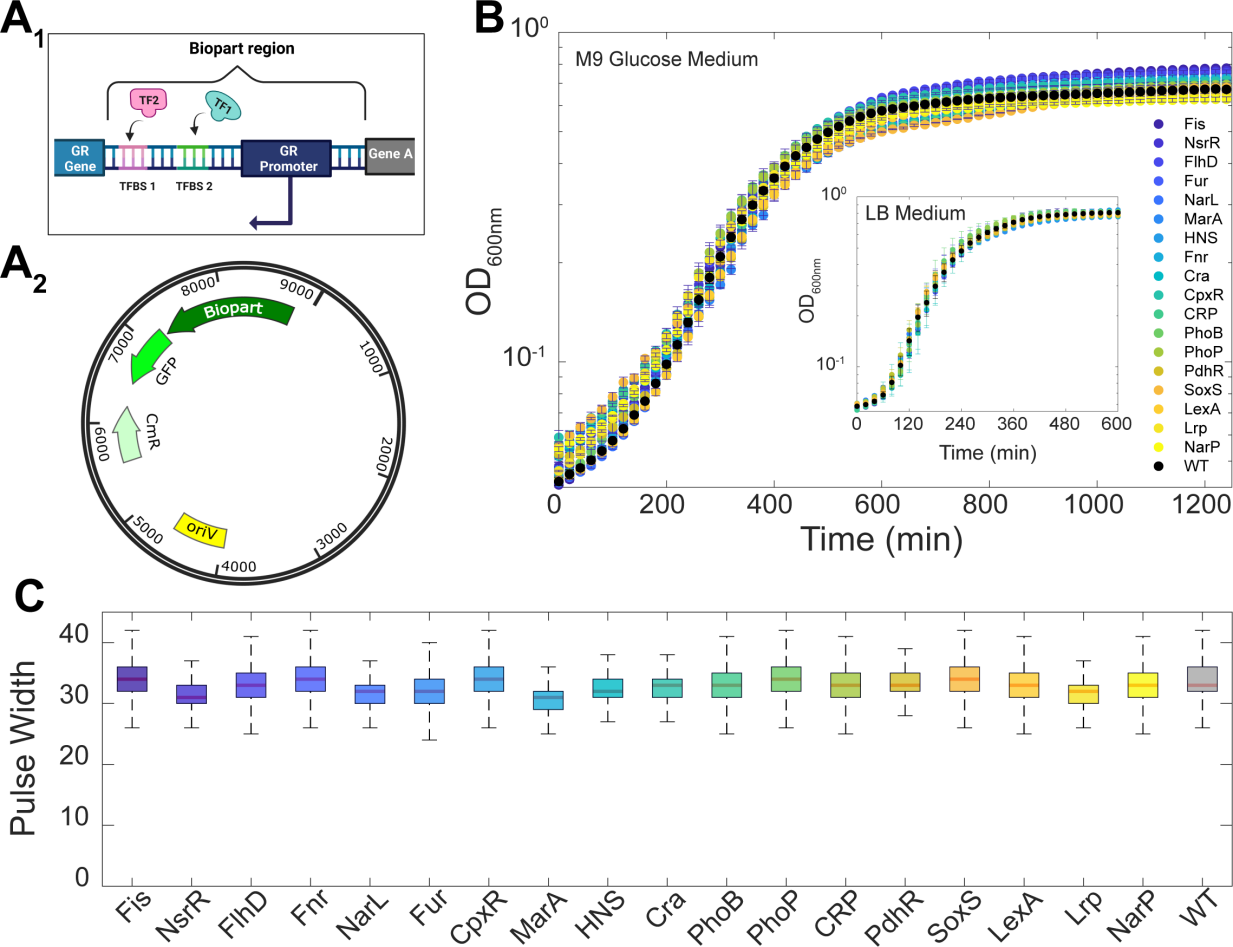
Structure of the plasmids and physiology and morphology of the strains carrying the GR reporters. (**A_1_**) Illustration of regions of the DNA from which bioparts were copied from. Each biopart is a copy of the non-coding region (promoter and TF binding sites), without the coding sequence of natural GR gene. Also shown are the start of the GR coding region, downstream from the biopart, and the end of an adjacent gene, upstream from the biopart, to assist in identifying the start and end of the biopart sequence. (**A_2_**) Illustration of the single-copy plasmid (pBAC) carrying the biopart (SnapGene, GSL Biotech). Shown are the regions coding for the biopart, the GFPmut3 downstream from the biopart, a chloramphenicol resistance gene (CmR), and the origin of replication (OriV). The figure was created with the assistance of BioRender.com. (**B**) Growth curves of the wild-type (WT) strain (MG1655) and of the strains carrying the plasmids coding for the GRs, respectively. The optical density at 600 nm (OD_600nm_) was measured every 20 min in minimal (M9 Glucose) medium at 37°C. The inset shows the OD_600nm_ in rich Luria-Bertani (LB) medium. The error bars are the standard error of the mean of three biological replicates. (**C**) Box plot of the average pulse widths from flow cytometry, used as a proxy for cell size, of each strain. The center of the boxes indicates the median. The bottom and top edges of the vertical line indicate the 25th and 75th percentiles, respectively.

We also collected the DNA sequences controlling the GRs expression profiles (from RegulonDB). These are the DNA sequences downstream of the previous gene coding region and upstream of the coding region of our GR’s gene of interest. Noteworthy, these regions include the promoters but do not include codes for any polypeptide. Moreover, no promoters are known to control GR expression outside of these regions. We thus used these DNA sequences as the “bioparts” (Fig. 2A1) of our GR reporters, which we designed on pBAC plasmids using SnapGene (GSL Biotech) (Fig. 2A2). As a side note, we failed to synthesize reporters for: IHF, ArcA, NtrC, and ModE. In detail, first, we failed to find promoter sequences for ModE and NtrC. Moreover, while we found the promoter sequences of IHF and ArcA, they formed stem loops during synthesis (due to the existence of complementary sequences), which hampered their integration into the plasmid backbones.

The plasmids (constructed in Integrated DNA Technologies, Iowa, USA) also include fluorescent protein-coding regions downstream of the biopart to report on the transcription rates in single cells (Fig. 2A). Specifically, we used single-copy pBAC plasmids containing GFPmut3 (18) (a kind gift from J. J. Collins). For a schematic representation of the backbone of this plasmid, see Data Availability.

GFPmut3 has a strong ribosome binding site to quickly produce many proteins from each RNA (18), and each protein is 21 times brighter than wild-type (WT) GFP (17), resulting in very strong signals. Moreover, GFPmut3 has a very fast maturation time [~4 min, Table 1 in reference (19)], entailing that its signal strength follows RNA levels with minimum delay. Furthermore, GFPmut3 has weak photostability (19), which mimics well the natural RNA degradation times of *E. coli* [~2–10 min (20)]. Finally, GFPmut3 is non-toxic, e.g., it does not form inclusion bodies and it does not react with natural cell components (14, 17), implying that it does not interfere with cellular functioning. All these features combined make GFPmut3 an efficient reporter of RNA levels over time (rather than of the number of proteins that the RNAs code for).

To confirm past reports informing that GFPmut3 is non-toxic, we studied growth in cells with and without the plasmid in minimal and rich growth medium, respectively (Materials and Methods section, “Bacterial strains and growth conditions”). In both media, we observed only small differences between our strains, including the WT (Fig. 2B). Moreover, we observed a relatively fast growth rate in Luria-Bertani (LB) medium.

We also did not find differences between cell sizes using pulse width (Fig. 2C) from flow cytometry as a proxy (21, 22) (Materials and Methods section “Flow cytometry”). Additionally, we also obtained cell sizes of eight strains, including the WT, by microscopy (Materials and Methods section, “Microscopy and image analysis”), and again did not find significant differences (Fig. S2A). Finally, we found no differences in cell viability (calculated as CFU per milliliter, where CFU stands for colony forming units) between the eight strains, including the WT (Fig. S2B).

**Fig 3:**
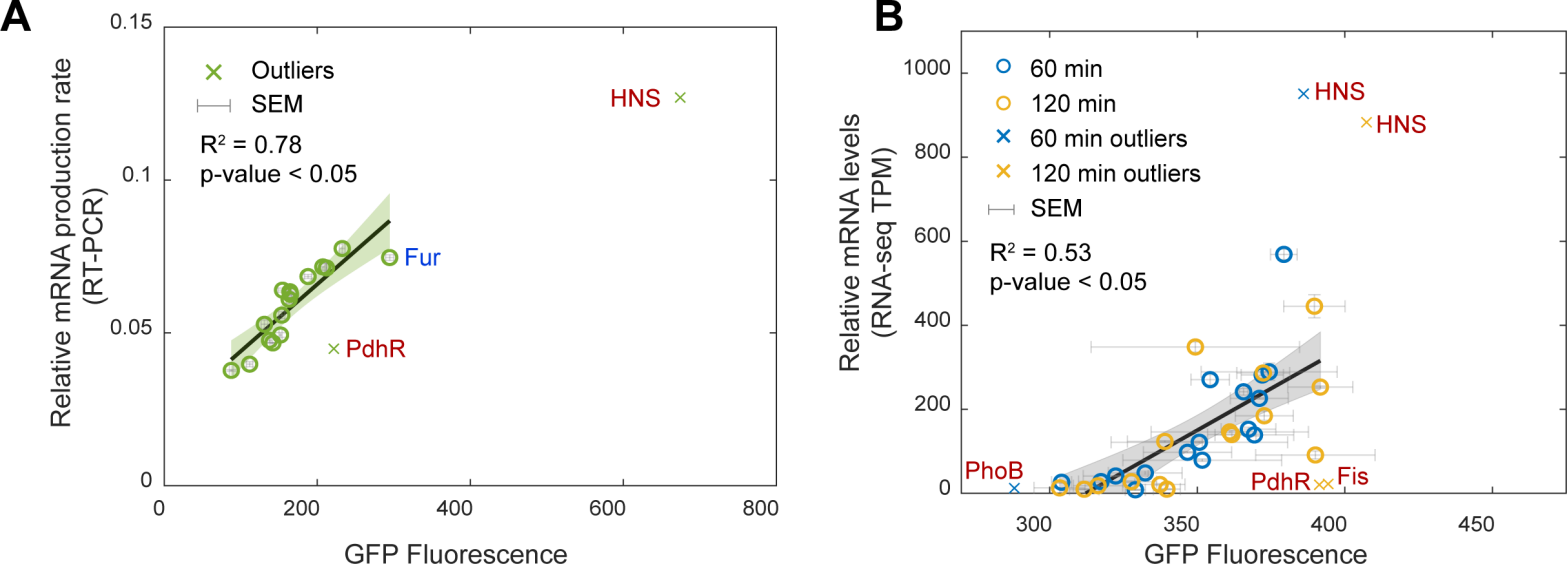
Correlation between RNA levels of the natural genes and the protein expression levels of the plasmids in standard (control) growth conditions. (**A**) Scatter plot between relative mRNA production rates of native GR chromosomal genes (Materials and Methods section, “RT-PCR”) and GFP levels of the corresponding reporter plasmids (Materials and Methods section, “Spectrophotometry”). The error bars are the standard error of the mean (SEM) from three biological replicates. Also shown are the *P*-value and the coefficient of determination, *R*^2^ (Materials and Methods section, “Fittings and statistical analysis”). The names of the outlier data points are shown in red, near the crosses. “Fur” is shown in blue (for details, see Table S3). (**B**) Scatter plot between Transcripts Per Million (TPM) normalized counts of GRs from RNA-seq (Materials and Methods section, “RNA-seq”) and the corresponding GFP. Measurements at 60 and 120 min after placing cells in fresh media (Materials and Methods section, “Spectrophotometry”). Error bars are the SEM from three biological replicates. Also shown are the *P*-value and the coefficient of determination, *R*^2^. The x-axis does not start at zero, for easier data visualization. The names of the outlier genes are shown in red, near the crosses.

Next, we tested if the promoters of the GR reporters behave similarly to the natural chromosomal-integrated GR promoters. For that, we confronted the average mRNA levels of the natural GR genes (Materials and Methods section, “RT-PCR”) with the average GFP levels from the GR reporter plasmids (Materials and Methods section, “Spectrophotometry”). We found a statistically significant linear correlation between them (Fig. 3A). However, 2 of the 18 probes (“HNS” and “PdhR”) can be classified as outliers (Materials and Methods section, “Fittings and statistical analysis”). Considering the remaining 16 probes alone, we find a high *R*^2^ for the linear fit (Fig. 3A). Moreover, as expected, the linear fit nearly intercepts the origin of the plot aside from a small deviation. Relevantly, that deviation is not significant since a linear fit crossing the origin still results in a fit that has both a high *R*^2^ and a *P*-value <0.05 (Fig. S3C). We concluded that the remaining 16 synthetic GR reporters are efficient.

To further test the efficiency of the GR reporters, we used RNA-seq data from reference (23) for the GR genes at two time moments during exponential growth (Materials and Methods section, “RNA-seq”). Then, we performed corresponding GRs’ GFP measurements by spectrophotometry in the same growth conditions and time moments. As above, we found a statistically significant linear correlation between the RNA-seq data and the average GFP levels from the GR reporter plasmids (Fig. 3B).

Interestingly, again, the probes of “HNS” and “PdhR” were outliers (in at least one of the two time points). Due to this confirmation, we did not include these two probes in our library of probes and did not consider them for further analysis. The few other probes classified as outliers were kept in the library since, neither were they outliers when using reverse transcription polymerase chain reaction (RT-PCR) nor in the two RNA-seq measurements (60 min and 120 min).

We additionally performed a final statistical analysis of the RT-PCR data (in Fig. 3A). Specifically, we considered the effects of removing each of the 16 remaining probes from the data, by assessing if any removal would significantly alter the *R*^2^ of the linear fit (Fig. 3A). The results in Table S3 show that only the removal of Fur would increase the *R*^2^ significantly. However, since Fur was not classified as an outlier in any of the three experiments (RT-PCR and the two RNA-seq time points), we opted for maintaining the strain carrying the probe for Fur in the library. Considering this, along with Fig. 3A and B, we concluded that the 16 GR reporters are all efficient reporters.

### GR reporter activities during the exponential and stationary growth conditions

We monitored the GRs’ transcription activity in exponential and stationary growth phases (Materials and Methods section, “Bacterial strains and growth conditions” and “Spectrophotometry”). To compare the activities between different GR probes, we normalized the raw population fluorescence values (Fig. S4A) by the corresponding optical density at 600 nm (OD_600_) values at the same time points (shown in Fig. 2B). This removed the influence from the size of the cell populations. The normalized values were used as a measure of the average single-cell fluorescence levels at each time point. We expect these values to differ between strains as they should reflect the expression dynamics of the various GRs.

**Fig 4:**
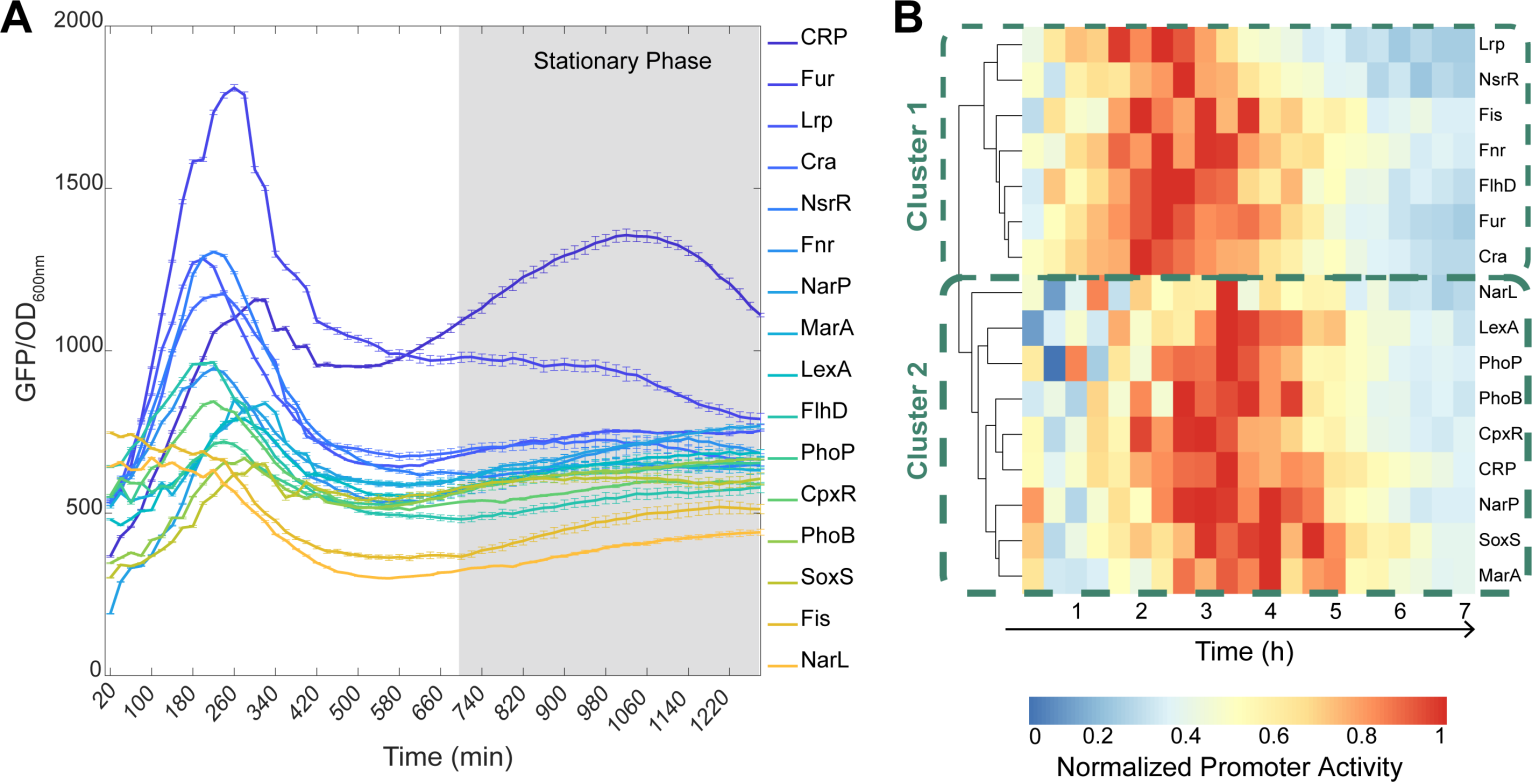
Transcriptional dynamics of the GR reporters in standard growth conditions. (**A**) Fluorescence levels normalized by the OD_600_ values over time of the strains carrying each of the 16 GR reporter plasmids during exponential and stationary growth phases. Cellular autofluorescence (obtained from the WT strain) was subtracted from all data points, but this had no significant influence on the results (Data Availability). The GR reporters in the legend are listed from top to bottom from the highest to the lowest fluorescence value in the y-axis at the minute 1,200, respectively. The gray area is the time window during which cells are in the stationary growth phase, which is preceded by the exponential growth phase. (**B**) Heatmap of the promoter activity ($\frac{\frac{d\left(GFP\right)}{dt}}{OD}$) of the reporters of the GRs over time. The data was produced using the R package “pheatmap” (version 1.0.12). The values shown are relative to the maximum level in each GR, to identify when it maximized. High and low values are colored red and blue, respectively (corresponding color bar in the top right corner). The dendrogram is shown on the left side of the heatmap. The GRs were compared using Euclidean distances and were clustered using the complete-linkage method.

From Fig. 4A, we found a wide variety of behaviors. For example, “CRP” and “Fur” exhibit much higher activity than “NarL” and “SoxS.” This is expected since “CRP” and “Fur” are commonly present during exponential growth (8, 24), while “NarL” and “SoxS” are induced during nitrate and oxidative stresses, respectively (25, 26). Meanwhile, “NarL” and “Fis” decrease, “SoxS” and “PhoB” are stable, and “NsrR” first increases and then decreases, among other diverse behaviors. We expect these differences to arise mostly from differences in RNA production dynamics alone, since all probes share the same RNA degradation rates and the same protein (GFPmut3) translation and degradation rates. The WT strain is not shown as the autofluorescence values are below 30 fluorescence units, which would not be visible (see Data Availability).

We also estimated the changes in GR promoter activities over time (Fig. S4B and Materials and Methods section, “Fittings and statistical analysis”). To facilitate comparison, we normalized the intensity levels of the GR reporters over time by their respective maximum expression level. From this, we obtained a heatmap, using the “pheatmap” R-package. Shown in Fig. 4B are the first 420 min, which is the period of time during which the promoters’ activity changes significantly (Fig. S4B). The “pheatmap” package estimates the difference in expression levels between two GR reporters from a Euclidean distance ($\sqrt{\Sigma_{i}{\left(x_{i}-y_{i}\right)}^{2}}$), where *x* and *y* are the expression intensity of each GR reporter, respectively. Meanwhile, *i* is the time moment (Table S4).

Next, the GR reporters were clustered based on these distances, using a complete-linkage method (pheatmap). The resulting dendrogram in Fig. S4C shows two dynamical behaviors. The first cluster (cluster 1) has genes whose promoter activity peaks at ~140 min (Lrp, NsrR, Fis, Fnr, FlhD, Fur, and Cra). Cluster 2 includes genes whose promoter activity peaks 60 min later (NarL, LexA, PhoP, PhoB, CpxR, CRP, NarP, SoxS, and MarA).

We also investigated gene ontology (GO) (27, 28) to identify which biological processes are the GR genes of the two clusters involved in. We compared their overrepresented biological processes (Fig. S5, Materials and Methods Section “Gene ontology”). Visibly, genes of cluster 1 are associated with positive regulation of biological processes. Meanwhile, genes of cluster 2 are associated to negative regulation of biological processes. Potentially, this could partially explain the differences in promoter activity.

As a side note, the fluorescence levels reported in Fig. 4A are affected not only by the transcription activity of the GR reporters but also by the degradation rates of the RNAs coding for GFP and by the GFP translation and degradation rates. Nevertheless, these three rates are identical in all strains. Consequently, the fluorescence intensities in Fig. 4A should only differ between strains, due to differences in the transcription rate of the GR reporters. Therefore, these intensities are a good proxy for the transcription activities of the GR promoters.

### GR reporters detect differences in single-cell variability of the GRs’ expression level

**Fig 5:**
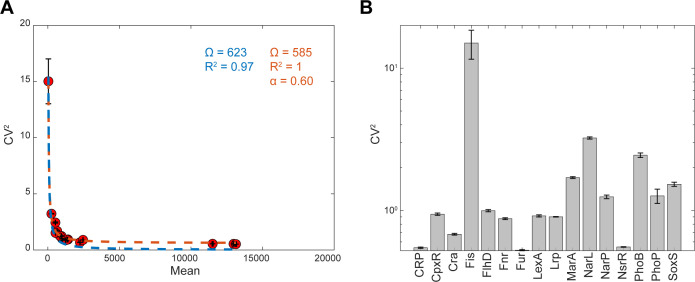
Single-cell expression levels of the GR reporters in standard growth conditions. (**A**) Scatter plot between the CV^2^ and the mean of the single-cell fluorescence for each strain. Shown are the best fitting lines ($CV^{2}=\frac{\Omega }{M}$) when (red line) and when not (blue line) considering a noise floor (α). The values shown are obtained from cells in the exponential phase (300 min, 0.3 OD_600_ in Fig. 4A). (**B**) CV^2^ of single-cell fluorescence levels of the strains carrying each GR reporter in (**A**). The y-axis is in log scale.

We measured the variability in the single-cell expression levels of the GR reporters of each strain. In Fig. 5A, we plotted the CV^2^ (squared coefficient of variation) against the mean, M, of the single-cell fluorescence levels (Materials and Methods section “Flow cytometry”). Then, in agreement with references (15, 29), CV^2^ best fits the data by the ordinary least squares method when assuming the function:


(1) $$CV^{2}=\frac{\Omega }{M}$$

where Ω is a constant (blue curve in Fig. 5A).

Unfortunately, this model fails to fit well the empirical data for high values of M (M > 2,000, Fig. 5A). Likely, this is because the model in (equation 1) does not capture the contribution from extrinsic noise sources (15, 29). These sources include variability in RNA polymerase (RNAP) and ribosome numbers. For example, recent studies reported that single-cell RNAP levels can differ by ~45% in cells growing in the same conditions, specifically in the exponential and stationary growth phases (16). We thus corrected the model by adding a constant *α* (representing a lower noise limit). This improves the fit, as measured by *R*^2^ (red curve in Fig. 5A):


(2) $$CV_{Corrected}^{2}=\frac{\Omega }{M}+\alpha$$

Note that ${CV}_{Corrected}^{2}$ is only an estimation of the ${CV}^{2}$ of the natural RNAs of the GRs. Specifically, we expect these natural RNA numbers of the GRs to be subject to different noise sources than the RNAs of the probes. For example, the natural genes are chromosome-integrated (unlike the reporters). Because of this, we expect positive supercoiling buildup to be influential in natural RNA numbers, but not in the RNA numbers of the probes (30).

Nevertheless, the reporters contain the entire promoter regions of the natural GRs. As such, they likely have inherited specific features of the dynamics of transcription of the natural promoters, controlling not only the mean rate of production (Fig. 3A and B) but also the single-cell variability in the RNA production dynamics (Fig. 5B).

To test this, we also measured the CV^2^ of single-cell protein levels of the two natural, chromosomal integrated GRs (Materials and Methods section, “Bacterial strains and growth conditions”) that are present in the YFP fusion library (15). Visibly, the single-cell variability in the expression levels of the “CpxR” gene is higher than of the “Fur” gene in both the chromosome-integrated YFP strains (four times higher, as measured by CV^2^) as well as when it is plasmid borne (two times higher). The difference in the relative increase could be due to different noise sources, e.g., positive supercoiling buildup. As a side note, we did not compare the CV^2^ of the natural genes (YFP strain library) and the reporters (presented here) of each GR, because the fluorescent proteins that they are tagged with differ.

### The GR reporters exhibit specificity and sensitivity to weak stresses

We considered the strains containing “Fur,” “MarA,” “SoxS,” and “LexA” GR reporters, which are responsive to stresses caused by an excess of iron, tetracycline, hydrogen peroxide (H_2_O_2_), and kanamycin, respectively (Table S2) (Materials and Methods section, “Bacterial strains and growth conditions”).

Specifically, first, “Fur” (ferric uptake regulator) binds to DNA and represses transcription in the presence of iron (31). This, evidence suggests, contributes to maintaining iron homeostasis (32). Adding excess iron to the media should increase the transcriptional activity of the GR reporter of Fur. Meanwhile, “MarA,” coded by the “marRAB” operon, controls the expression of genes involved in the resistance to antibiotics, including tetracycline (33). Thus, tetracycline should induce the GR reporter of MarA (it would be possible to use aTc instead, if at lower concentrations). Similarly, oxidative stresses, caused by an excess of H_2_O_2_, damages *E. coli* cells (34). One gene known to be induced by H_2_O_2_ is “SoxS,” which regulates the expression of several genes (26). We should observe an increase in SoxS GR reporter’s activity under oxidative stress. Finally, we expect kanamycin to increase the activity of the GR reporter of “LexA,” since kanamycin causes DNA damage (35), which activates the SOS response pathway (36).

We measured, using spectrophotometry, the time-lapse response intensities of our reporters for “Fur,” “MarA,” “SoxS,” and “LexA” to all four stresses above, respectively. We first tested weak stress conditions, i.e., not strong enough to affect cell growth rates significantly (Fig. S7). For this, we started by normalizing the fluorescence values of the genes at each time point by the corresponding OD_600_ values of their strains at the same time point (i.e., GFP/OD). Then, we calculated the fold-change (FC) in these normalized values, for each GR probe, in each stress condition. For this, we divided these values by the corresponding values when in standard growth conditions (reported in Fig. 4A).

**Fig 6:**
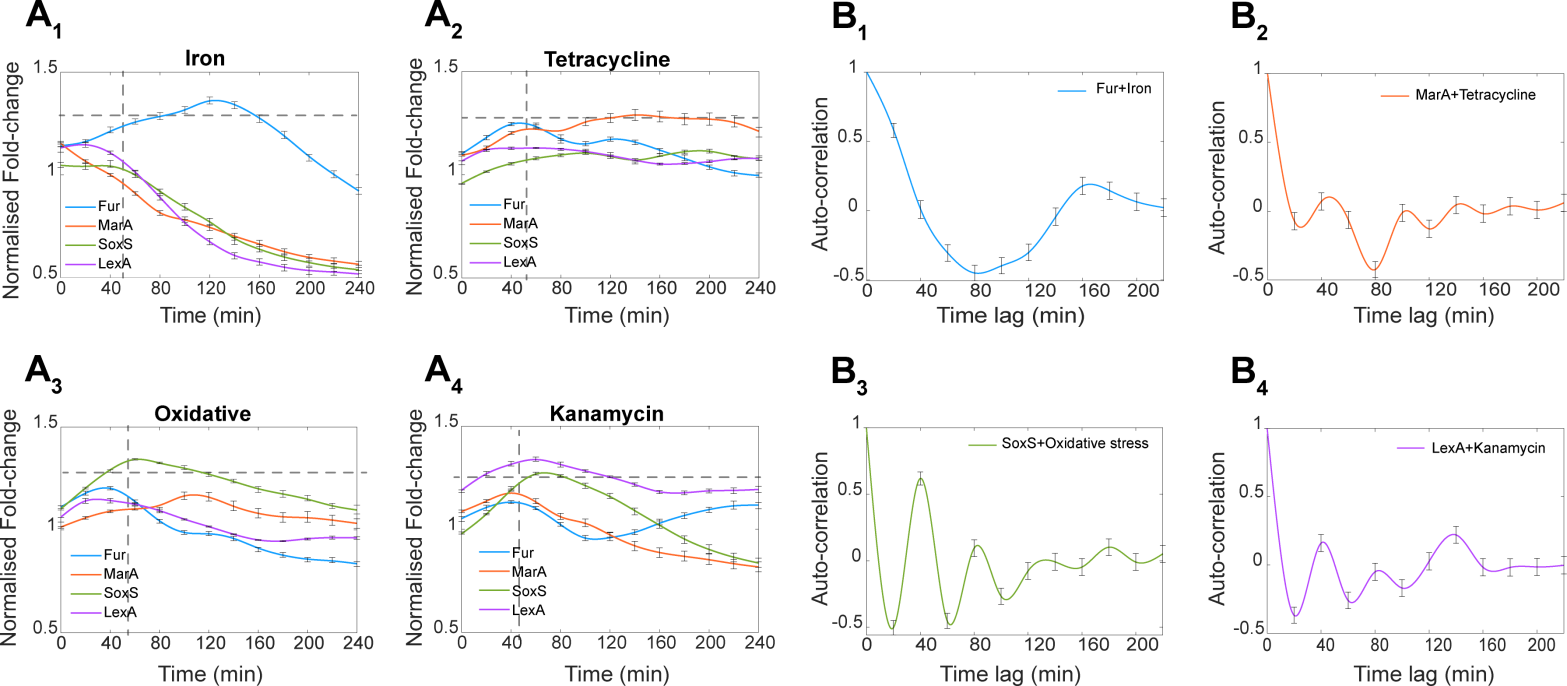
Activities of the GR reporters under weak stress conditions. (**A**) GR reporter levels over time when under weak stresses. Levels are relative to the standard growth condition in the same time point. Shown are the stresses: (**A_1_**) iron excess (0.2 µM), (**A_2_**) tetracycline (0.05 µg/mL), (**A_3_**) oxidative stress (0.6 mM H_2_O_2_), and (**A_4_**) kanamycin (25 µg/mL). The error bars are the SEM of three biological replicates. Cellular autofluorescence (obtained from the WT strain) was subtracted from each data point, including the standard growth condition. Values above 1 correspond to overexpression, while values below 1 correspond to repression, when compared to the control. The horizontal dashed lines signal the value above which the fold-change is high enough to be classified as “true positive.” Meanwhile, the vertical dashed lines correspond to the time after which we quantify sensitivity and specificity (40 min). (**B**) Autocorrelation of the time-lapse mean expression levels of the GR reporters, when under their respective stress. (**B_1_**) iron excess, (**B_2_**) tetracycline, (**B_3_**) oxidative stress, and (**B_4_**) kanamycin. The error bars correspond to the SEM of three biological repeats.

This allows comparing the transcription rates of the GRs. A normalized FC of a GR during the stress will be either 1 (in case of no change) or more or less than 1 in case of overexpression/inhibition, respectively. We only used data from 50 min onward after placing cells under stress to account for protein production times in *E. coli* (Materials and Methods section, “Response times of the GR reporters”). From Fig. 6A1–4, each GR reporter responds effectively to its specific stress, and only that one.

From these data, to quantify the reporters’ efficiency, we calculated their sensitivity and specificity (Materials and Methods section, “Sensitivity, specificity, and fitness”), i.e., their capacity to detect the true signal (sensitivity), while not responding to other signals (specificity), respectively. For this, we define a threshold for the FC, above which the signal is classified as “responsive,” while below it is “non-responsive.” Specifically, we tested lower and upper bound threshold values of 1.0 and 1.3, respectively (Table S5). A threshold of 1.3 mildly maximizes the fitness (Materials and Methods section, “Sensitivity, specificity, and fitness”), defined as being the sum of the average sensitivity and average specificity of the four reporters to the four stresses (Fig. S6B). Nevertheless, the fitness is not very influenced by the threshold, which suggests that the reporters are functionally robust. Overall, given the high sensitivity and specificity (Table S5), we conclude that the reporters are efficient under weak stress conditions. As a side note, during iron excess, the reporters for “MarA,” “SoxS,” and “LexA” responded negatively. This is expected, as noted above, and does not affect the sensitivity and specificity.

### The GR reporters can detect negative autoregulation mechanisms

Out of the 22 GRs studied, 5 have known positive autoregulation mechanisms, while 8 are known to be subject to negative autoregulation (2). These mechanisms cause distinct behaviors (e.g., negative autoregulation usually causes oscillations, within certain ranges of parameter values). We tested whether oscillations can be detected by the probes of “Fur,” “SoxS,” and “LexA,” which are negatively autoregulated. As a control, we also evaluated “MarA,” which is positively autoregulated (2). Thus, it should not oscillate.

We searched for oscillations in the time-lapse responses to the weak stress conditions (Fig. 6A1–4) (Materials and Methods section, “Fittings and statistical analyses”). From Fig. 6B1–4, we find oscillations in “Fur” (80 min long), “SoxS” (40 min long), and “LexA” (40 min long). Meanwhile, we did not find it in “MarA.” We conclude that the probes detect negative autoregulation, when existing.

### The GR reporters maintain their specificity and sensitivity under strong stresses

We studied the behavior of the reporters in cells under strong stresses. We classified a stress as “strong” if the growth rate differs from the standard growth condition. The protocols to impose the stresses are described in the Materials and Methods section “Bacterial strains and growth conditions.” Since these stresses affected the growth rates (Fig. S8A_1_), we opted for not using spectrophotometry to measure the activity of the reporters. Instead, we used flow cytometry to measure reporter activities at the single-cell level. As a side note, in agreement with past reports (37, 38), we found faster growth under strong iron excess.

Growth rates have been related with cell size during standard growth conditions and, thus, could be related with intracellular protein levels (39, 40). Thus, here, we first investigated the relationship between average cell size and single-cell fluorescence, under the strong stresses. From Fig. S8A_2_ and S8A_3_, respectively, both are disturbed by the stresses. Thus, we searched for correlations between average cell size, average cell fluorescence, and average cell growth rates of the strains under standard and stress conditions. We found no correlations (Fig. S8B_1_, S8B_2_, and S8B_3_; see also Fig. S6A and S6C). We also found no correlations when subjecting each strain to the other stresses (Fig. S6D). Moreover, at the population level, the differences in mean expression levels of the GR reporters shown in Fig. S8A_3_ are not, on average, related to changes in mean cell size and/or in mean growth rate. Instead, as expected, they are likely the outcome of the GR reporter’s responsiveness to the stresses.

**Fig 7:**
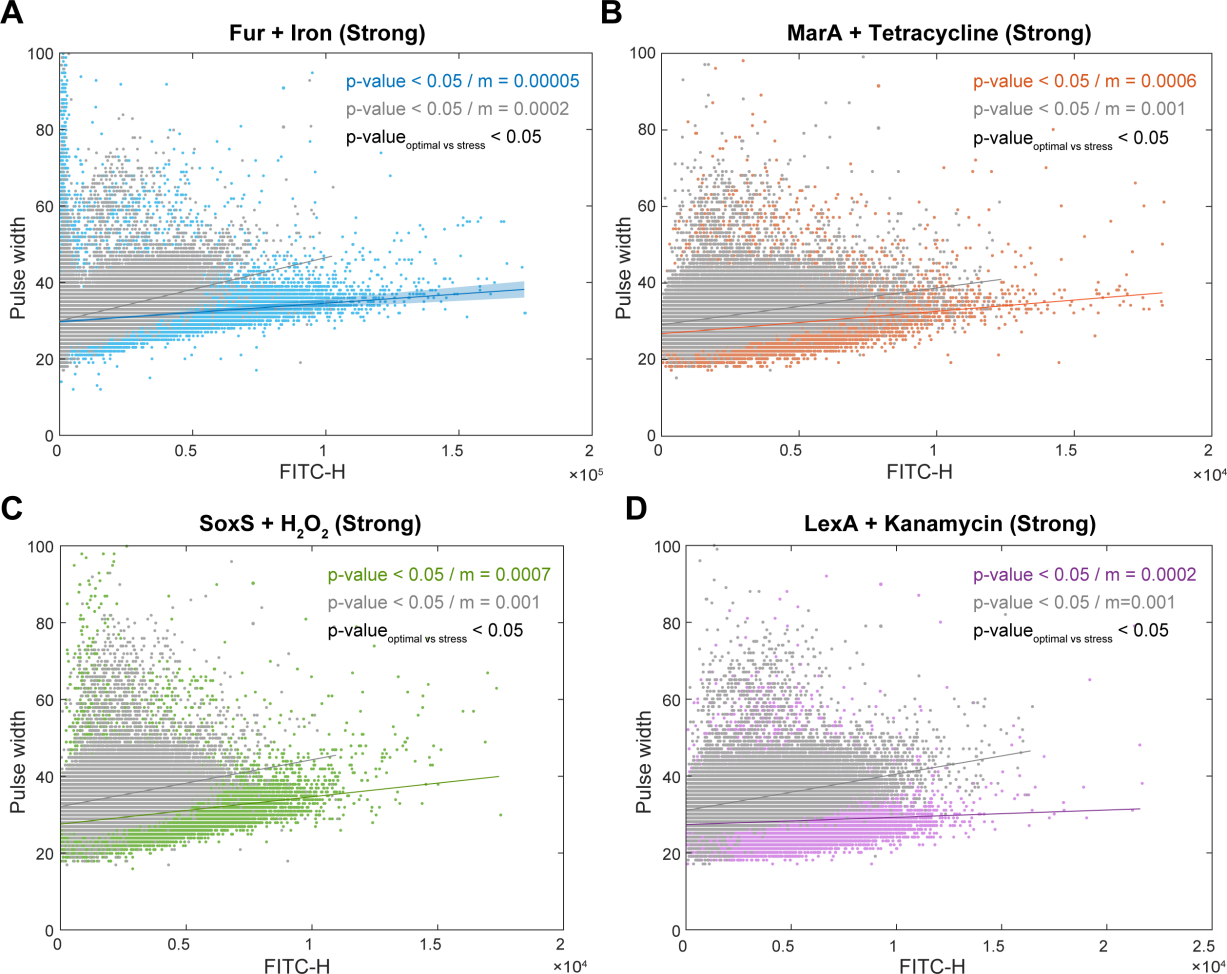
Single-cell size and GR reporter activities under the specific strong stresses. Measurements under (**A**) iron excess (2 µM), (**B**) tetracycline (5 µg/mL), (**C**) oxidative stress (5 mM of hydrogen peroxide), and (**D**) kanamycin (50 µg/mL). Single-cell expression levels (FITC-H) are plotted against the pulse width (a proxy for cell size). Colored dots are cells subject to the stress. Gray dots are cells in standard growth conditions, shown for comparison. Also shown are the best-fitting lines to each of the two cohorts. The *P*-value of each line is from a statistical test of whether the linear fit differs from a horizontal line, using “fitlm” in MATLAB. For *P*-value >0.05, we cannot conclude that the line differs from a horizontal line. Measurement time corresponds to 120 min in Fig. S8A_1_.

Next, we evaluated the reporters’ sensitivity at the single-cell level, after 120 min under the strong stresses (Materials and Methods section, “Sensitivity, specificity, and fitness”). From Fig. 7A through D, both in standard growth and in strong stress conditions, there is a positive, statistically significant correlation between the reporters’ fluorescence intensity and the pulse width. This is expected since these fluorescence proteins are decaying largely due to cell division (i.e., dilution). However, the fluorescence levels are stronger under stress [specifically, the average FITC-H (fluorescein isothiocyanate detection channel) are higher by 1.3 times, 1.5 times, 1.4 times, and 1.3 times, respectively]. This causes the inclination of the best-fitting line to be ~10 times smaller in stress conditions (Fig. 7A through D). As expected, the *p*-value comparing the slopes (using a *t*-test statistics) is smaller than 0.05, from which we conclude that the two slopes are not from the same distribution. Overall, we find that all reporters were sensitive to the strong stresses, with their response not being affected by small differences in average cell sizes between the strains.

Finally, we assessed the reporters’ “specificity” (Materials and Methods section, “Sensitivity, specificity, and fitness”) by subjecting the strains to strong stresses, other than the stress they should detect. Their response strengths were weak in all cases, causing the relationship between pulse width and cell fluorescence to differ little from when in standard growth conditions (Fig. S9). In agreement, in general, the inclinations of the best fitting lines for standard growth and for “other” stress conditions are of the same order of magnitude. Also, as expected, average pulse widths and average cell fluorescence of the strains remain uncorrelated, when subject to these other stresses (Fig. S6D).

### Robustness of the relationship between GFP-mRNA and GFP-protein levels of the probes

At least four cellular processes influence the ratio between RNA and protein levels in a cell: translation, RNA degradation, protein degradation, and cell division [which causes not only dilution but also, in some cases, asymmetries when partitioning proteins between sister cells (41–43)]. If the rate of any of these processes changes, the ratio between RNA and protein levels can change, unless another rate(s) also changes, in a manner that compensates for the first change.

Evidence suggests that the ratio between RNA and corresponding protein levels can differ between conditions in *E. coli*. For example, under amino acid starvation, ribosome concentrations can decrease significantly, reducing the average number of proteins produced from each RNA (44). Similarly, for example, during oxidative stress, protein degradation is accelerated (45), whereas during heat shocks, the RNA degradation is accelerated (46).

We estimated the robustness of the ratio between GFP-RNA levels and corresponding GFP levels produced from the probes by performing RT-PCR of GFP mRNA levels (Materials and Methods section, “RT-PCR”) under the stresses whose GFP levels were studied above (Fig. 7). Fig. S10 shows how GFP-protein levels follow GFP-mRNA levels. Arguably, a curve fits the data better than a line (it has higher *R*^2^). This suggests the existence of small differences between stresses in the ratio between RNA levels and protein levels.

Nevertheless, all data points (when accounting for the uncertainty) are within the 95% confidence intervals of the linear fit. As such, there is a sufficiently robust linear relationship between RNA and protein levels, to allow comparing of the expression levels of the GRs between the stresses studied here, without quantitative adjustments.

Meanwhile, for example, in reference (47), it was shown that some changes in media composition can alter growth rates in a manner that will alter the relationship between RNA and protein levels. Similarly, this relationship can differ also between, e.g., growth phases. In such cases, if one was to use our GR reporters to compare GR levels between conditions, quantitative adjustments would be needed.

## DISCUSSION

The TFN of *E. coli* has evolved an out-degree distribution that follows a power law (23), due to which a small fraction of the genes expressing TFs (22 of 216) directly regulate many genes [~31% of all ~4,747 genes in the genome (RegulonDB 11.1)] (2). Following the RNA and/or protein levels of each of these GRs is, thus, a key step for dissecting the transcriptional programs of stress response. With this goal, we engineered a library with 16 out of these 22 GR reporters.

We showed that the single-copy plasmids coding for the reporters, under the control of copies of the natural GR promoters, do not affect significantly neither the morphology nor the physiology of the host cells. Additionally, due to the fast maturation times of the GFP coded, the GR reporters track well the native RNA levels of the GRs. As such, the GR reporters can sense changes in the global transcriptional programs a few minutes after these emerge. This is important since, in *E. coli*, most regulatory mechanisms of gene expression act on transcription (48–50).

Prior to this library, reporters have been engineered for only a few GRs. Also, they differ in their rates of protein folding and degradation, in photo bleaching, etc., hindering direct comparisons (and in assessing how these rates change with environmental conditions, etc.). Our library is the first to allow direct comparisons of the dynamics of most GRs due to using the same fluorescent reporter and WT strain to engineer all strains.

One expected application of this library is the study of the temporal levels of the GRs during life-threatening, genome-wide stresses affecting hundreds of genes. This may allow explaining how the gene regulatory network (GRN) of *E. coli* carries ordered, short- and long-term transcriptional programs involving large gene cohorts that introduce advantageous phenotypic changes while robustly maintaining homeostasis.

Moreover, since the reporters are in plasmids, it will be easy to combine them with other synthetic libraries. For example, combining our GR reporters with strains from the KEIO collection (51) and the ASKA library (52) should allow assessing how much a given gene influences the GRs. Similarly, a “Tunable TF library” (53) was recently produced to synthetically tune the concentration of most TFs. However, it cannot monitor natural production rates. Combining our reporters with this library, one can assess how TFs can affect GR levels, etc.

Interestingly, while the GR reporters have the native promoter sequences (including operator sites), they all exhibited similar response times to the various stresses, as well as to the transition to stationary growth phase. While this is enhanced by all reporters coding for the same GFP protein, it also requires similar transcription rates. As such, our results suggest that there may exist an evolved synchrony in response times between GRs that may be critical for the proper functioning of complex transcriptional programs involving hundreds of genes.

In the future, to enhance this library’s ability to reproduce the temporal numbers of natural GRs, we could introduce their native ribosome binding site (RBS) sequences in front of the GFP coding sequence. We could also use mutant GFP sequences with different production times to account for different protein folding times of the GRs. Moreover, we could use tandem promoters to further fine tune GFP production (54), among others.

Finally, we expect this new library of GR reporters to have wide applicability in synthetic biology, therapeutics, and bioindustrial efforts involving *E. coli*. For example, the library could be used to investigate if and how GRs are involved in the loss of functionality that synthetic circuits usually suffer with changing external conditions. Second, we can use the library to identify GRs (and subsequent transcriptional programs) involved in bacterial tolerance to antibiotics, which is a critical step for the emergence of resistance. Specifically, we can now track GR levels, to find which ones may be involved in enhancing tolerance. Finally, this library can be useful in bioindustrial processes. Currently, many bioindustrial processes are energetically costly. For example, several bioindustrial processes need to be carried out at high temperatures. Potentially, by modifying key transcriptional programs, it may be possible to engineer strains that require lesser extreme temperatures, albeit at the cost of some survivability. Tracking GR levels is a necessary step for identifying which GRs need to be tuned to adjust those cellular decisions.

## MATERIALS AND METHODS

### Bacterial strains and growth conditions

We used *E. coli* K-12 MG1655 cells (WT strain), as it is also used in references (14, 15, 53). Chemically competent (CC) *E. coli* K-12 MG1655 cells were prepared for plasmid transformation. For each strain, 5 µL of the plasmid DNA (10 ng) coding for the GR reporter was mixed with 50 µL of MG1655 CC (1:10 ratio), and then incubated on ice for 30 min. Next, we kept the mixture at 42°C on a water bath for 1 min. Finally, 800 µL of super optimal broth with catabolite repression medium (SOC) medium was added to the mixture, which was kept at 37°C under aeration at 250 RPM for 1 hour.

From each mixture, 200 µL was plated using the spread plate method on fresh LB agar plates (2%), prepared by supplementing with antibiotics (34 µg/mL chloramphenicol). Finally, the plates were kept overnight at 37°C. The next day, three colonies were picked from each plate and inoculated in fresh LB medium, supplemented with antibiotics (34 µg/mL chloramphenicol). Afterward, the cells were incubated at 30°C overnight with shaking at 250 RPM. The resulting culture cells were diluted into fresh M9 media (0.03 optical density, i.e., OD_600_) and were supplemented with amino acids, vitamin solutions, and 0.4% glucose as the carbon source.

In general, measurements were conducted using overnight culture cells. The control condition was M9 medium at 37°C. On average, the mid-exponential phase was reached in 300 min (OD_600nm_ ~0.3). At this stage, for each of the 18 strains, we performed various measurements of GR reporter activities and other control measurements using RT-PCR, colony counting for assessing cell viability, flow cytometry, and microscopy.

For weak stress conditions, upon reaching OD_600nm_ ~0.3, four strains containing “Fur,” “MarA,” “SoxS,” and “LexA” plasmids, respectively, were subject to iron, tetracycline, kanamycin, and H_2_O_2_, respectively. To subject cells to excess of iron, M9 medium was supplemented with iron citrate, as in reference (55). A stock solution of iron citrate (iron-to-citrate ratio, 1:100) was made by dissolving ferrous sulfate (4 mM) in sodium citrate (400 mM). The pH was adjusted to 7 with NaOH. Finally, 0.2 µM of iron citrate was supplemented to the growth medium. Meanwhile, tetracycline and kanamycin were added to the growth medium in the concentrations of 0.05 µg/mL (56) and 25 µg/mL (35), respectively. Moreover, oxidative stress was induced by adding 0.6 mM H_2_O_2_ to the solution. For this, we diluted H_2_O_2_ solution from Merck Life Science (originally at 30%) until it reached 6%, as described in reference (57).

Similarly, for strong stress conditions, the same four strains, upon reaching OD_600nm_~0.3, were subject to iron (2 µM), tetracycline (5 µg/mL), kanamycin (50 µg/mL), and H_2_O_2_ (5 mM H_2_O_2_), respectively. Flow cytometry measurements were taken 120 min afterward. Finally, we also diluted overnight culture cells into fresh LB media to monitor the growth as well as to measure their GFP levels after 60 and 120 min. The latter was compared to the RNA-seq data from reference (23).

### Spectrophotometry

We measured the optical density at 600 nm to monitor the cell growth, using a Biotek Synergy HTX Multi-Mode Reader. With the same machine, we measured GFP fluorescence of cell populations over time. For this, the solution containing the cells was excited at 485 nm, while the emission was recorded at 528 nm.

### Microscopy and image analysis

To prepare cells for microscopy, first, the cells were pelleted and re-suspended in ~100 µL of the media. Then, 3 µL of cell suspension was placed on a 2% agarose gel pad made up of M9 medium and kept in between the round microscope slide and a coverslip. It took less than 5 min to move cells from the incubator to the microscope and start the observation. This time gap includes the assembly of the microscope imaging chamber containing the slides with the cells. Phase-contrast images were taken by an external phase-contrast system, and then were analyzed by the “CellAging” software (58) to automatically segment cell borders and extract cell areas. We used the areas as proxies for cell size.

### RT-PCR

Cells were harvested from three independent colonies in the mid-exponential phase (0.3 OD_600nm_). Then, 5 mL of the culture was immediately treated with a double volume (10 mL) of RNAprotect Bacteria Reagent (Qiagen, Germany) for 5 min at room temperature, to prevent RNA degradation. Next, enzymatic lysis was performed with Tris-EDTA lysozyme (15 mg/mL) buffer (pH 8.3). From the lysates, the RNA content was isolated using RNeasy purification kit (Qiagen) as per the manufacturer instructions. The RNA yield (~2 µg/µL) and absorbance ratios A_260_/A_280 nm_ and A_260_/A_230 nm_ were measured by a NanoDrop 2000 Spectrophotometer (Thermo Fisher Scientific, USA). The ratio was found to be 2.0–2.1, which indicates highly purified RNA.

Afterward, we removed DNA contamination. For this, samples were treated with DNaseI (Thermo Scientific, USA) as per the manufacturer instructions. The cDNA was synthesized from 1 µg of RNA using iScript Reverse Transcription Supermix (BioRad, USA) as per the manufacturer instructions. Next, cDNA samples (10 ng/µL) were mixed with qPCR Master Mix with iQ SYBR Green Supermix (BioRad, USA) with primers (10 µM) for target and reference (16S rRNA) gene, respectively.

Finally, we analyzed the samples using a BioRad MiniOpticon Real-Time PCR System (BioRad, USA). The thermal cycling protocol was 40 cycles of 95°C for 10 s, 52°C for 30 s, and 72°C for 30 s, with the fluorescence being read after each cycle. qPCR efficiencies of these reactions were >95%. No-reverse transcriptase (RT) and no-template controls were used to crosscheck for non-specific signals and contamination. Cq values from the CFX Manager Software were used to calculate the fold-change in the target genes (after subtracting for the reference gene), using Livak’s 2^-ΔΔCT^ method (59).

### Flow cytometry

We measured single-cell fluorescence using an ACEA NovoCyte Flow Cytometer (ACEA Biosciences Inc., San Diego, USA). Cells were diluted (1:10,000) into 1 mL of phosphate buffer saline solution, vortexed for 10 s. For each strain, we obtained three biological replicates (50,000 cells each). The flow rate was set to 14 µL/min. The data were collected by a NovoExpress Software v.1.6 (ACEA Biosciences Inc.).

To detect GFP and YFP, we used a blue laser (488 nm) for excitation. For emission, we set a core diameter of 7.7 µM and a photomultiplier tubes' (PMT) voltage of 600 and we obtained the values of the fluorescein isothiocyanate detection channel (530/30 nm filter) and of side scatter height and FSC-H (forward scatter height).

We set two lower bounds (detection thresholds), one for FSC-H (set to 5,000) and another for FITC-H (we removed the 1% highest values). These bounds removed any significant interferences from the data. Noteworthy, we never detected significant differences between the three repeats in any strain. We also measured the pulse width, which we used as a proxy for cell size (21, 22).

### RNA-seq

The RNA-seq data used were first published in reference (23). Briefly, overnight cultured cells were diluted into fresh LB media. RNA-seq measurements were performed 60 and 120 min after that. From the raw data in (23), RNA sequencing reads were trimmed to remove adapters and low-quality reads by Trimmomatic v.0.36. Quality check was done using fastqc. Trimmed reads were then aligned to the reference genome of *E. coli* MG1655 (NC_000913.3) using Bowtie2 aligner v.2.3.5.1. Unique gene hit counts were calculated with featureCounts from the Rsubread R package (v.1.34.7). Genes with less than five counts in more than three samples were removed from further analysis. The relative abundance of mRNA in each condition was calculated using transcripts per million normalization (60).

### Fittings and statistical analysis

To find best fitting lines (e.g., Fig. 3A and B), we used linear regression functions (MATLAB). Goodness of fit was estimated from *R*^2^ values. Meanwhile, we used the *t*-statistic with the null hypothesis that the slope of the best fitting line (of a scatter plot between the variables) is not different from zero. For *P*-values smaller than 0.05, we rejected the null hypothesis. Also, we used an iterative procedure to discard outliers in linear correlations (14) (Fig. 3A and B). The outliers were classified as data points beyond the boundaries of ellipse that encompassed 90% of the data (Fig. S3A and B).

For calculating a GR promoter activity [as described in reference (14)], OD and GFP curves were time shifted, so that all OD curves reached 5% of their maximal OD at the same time after background subtraction. Each background GFP (WT) value was then subtracted from the reporter strain GFP value at the same OD (and not necessarily at the same time point). Expression profiles were calculated by dividing GFP by OD. Then, promoter activity was calculated by taking the time derivative of GFP/OD between consecutive time points ($\frac{\frac{d\left(GFP\right)}{dt}}{OD}$) . All analysis steps were performed automatically using MATLAB Software. The standard error of the mean for FC analysis was calculated using an error propagation method, using the formula: $SEM=\frac{Mean_{2}}{Mean_{1}}\sqrt{\frac{{\left(SEM_{1}\right)}^{2}}{{\left(Mean_{1}\right)}^{2}}+\frac{{\left(SEM_{2}\right)}^{2}}{{\left(Mean_{2}\right)}^{2}}}$. Here, 1 and 2 represent the two groups compared (e.g., stress and control, respectively).

Finally, we searched for the existence of oscillations (an expected outcome of negative gene expression autoregulation) in spectrophotometry data. For this, we applied the “detrend” function (MATLAB) to the average cell population fluorescence levels normalized by population size (OD). “detrend” removes (here, by subtracting) the effect of a best straight-fit line from the data caused by, e.g., the increase in total cell fluorescence due to increasing cell population size. Then, we apply the “xcov” function to the treated data, to calculate the autocorrelation as a function of the lag, at each time point.

### Gene ontology

To study the GO representation (27, 28) of different clusters of GR genes, we performed an overrepresentation test using the PANTHER Classification System (61). This test finds statistically significant overrepresentations using Fisher’s exact test, which rejects the null hypothesis that there are no associations between the genes’ cohort and the corresponding GO of the biological process for *p*-values <0.05. This *p*-value is corrected for the false discovery rate using the Benjamini-Hochberg procedure (62). In case of biological processes that were overrepresented in both clusters, we opted to classify them in the cluster that has a greater fold enrichment, i.e., the cluster that has the most GR genes associated to a biological process than expected by random chance.

### Response times of the GR reporters

The GR reporters (Table S2) operate by producing fluorescent protein GFP at a certain rate once the promoter that they carry is activated by the respective signal. This production is preceded by several events, which take a significant amount of time.

First, once the substance to report (e.g., antibiotic) is introduced in the media, it quickly becomes homogeneously distributed (because the media is under constant mixing). Then, these molecules need to reach the cell cytoplasm and the promoter of interest within. For this, they need to cross the periplasm, and interact with the promoter controlling the expression of the GR reporter. Past studies suggest that substances such as Isopropyl β- d-1-thiogalactopyranoside (IPTG) and arabinose can take, on average, 20–40 min to be in sufficient numbers inside the cytoplasm to activate/repress the target promoters efficiently (63, 64). We assume an average time length of 30 min, for simplicity, for all substances tested here.

Next, once the promoter of the reporter is active, we expect transcription elongation of the RNA coding GFP to be a relatively fast process. Specifically, the DNA sequence coding for GFPmut3 is 717 nucleotides long (18). Meanwhile, transcription elongation should occur at approximately ~42 nucleotides /s (65). Thus, elongation should take ~15–20 s. Finally, it follows translation and maturation of the reporter protein, GFPmut3, which is ~4 min (19).

Given the above, we estimate that our GR reporter, if its promoter is sensitive to the substance, will have already increased sufficiently in levels approximately ~50 min, after the target substance is added to the media.

### Sensitivity, specificity, and fitness

To define the sensitivity, specificity, and fitness of the GR reporters, first, we considered the data from spectrophotometry (Fig. 6A1–4). For weak stresses (which do not disturb growth rates or cell sizes), GFP and OD measurements were conducted using spectrophotometry. We define that if the fold-change between two conditions in the expression level of a GR reporter is above a specific threshold, then the signal is “positive.” Else, if the fold-change is equal or below the threshold, then the signal is negative.

Moreover, we classify signals as “true” or “false.” For this, we use Table S2. For example, the GR “Fur” should respond to iron excess. Also, Fur should not respond to the other stresses tested (excess of H_2_O_2_, tetracycline, and kanamycin), since there is no past evidence of such responsiveness. Given this, if the reporter for Fur responds positively to iron excess, we will classify its signal as “true positive” (TP). If the response is negative, we classify its signal as “false negative” (FN). Meanwhile, if it responds positively to another stress, we classify that signal as false positive (FP). If the response is negative, we classify that signal as true negative (TN).

Given this, the “sensitivity” of a GR reporter is defined as its capacity to detect true signals. Formally, it can be quantified by a “true positive rate,” which is the number of TP findings over the number of all positive (P) findings (true positive plus false positive findings):


(3) $$TPR=\frac{TP}{P}$$

When having time series data on a reporter from a spectrophotometer, we apply this formula to each time moment, and then obtain the average over all time moments, *t*, as follows:


(4) $$TPR\left(t\right)=\sum_{i=1}^{i=N\left(t\right)}\frac{TP_{\left(i\right)}}{P_{\left(i\right)}}$$

Meanwhile, “specificity” of a reporter is its capacity to remain unresponsive when there is no true signal to detect. In general, specificity is quantified by a “true negative rate,” which is the fraction between TN and total negatives (N), i.e., true, and false negatives:


(5) $$TNR=\frac{TN}{N}$$

Having information on several time points from a spectrophotometer, one can use the formula:


(6) $$TNR\left(t\right)=\sum_{i=1}^{i=N\left(t\right)}\frac{TN_{\left(i\right)}}{N_{\left(i\right)}}$$

Finally, we define fitness as the average between the sensitivity and specificity. Assuming several time points, it follows:


 (7) $$Fitness=\frac{\sum_{i=1}^{i=N\left(t\right)}TPR_{\left(i\right)}+TNR_{\left(i\right)}}{N\left(t\right)}$$

For flow cytometry data, there is only one time point. Moreover, the definition of TP, TN, FP, and FN differ. In this case, we compare the inclinations of the best fitting lines to the scatter plots between FITC-H (a proxy for transcription rates) and pulse width (a proxy for cell size).

Assume an inclination *m_o_* for a population of cells carrying the plasmid responsive to stress *x*, when in the standard growth condition. Now, assume an inclination *m_x_* for cells under the “correct” stress condition. Notably, if the reporter is to be responsive to a stress, FITC-H value should increase. Meanwhile, pulse width should not differ or differ little. This will cause *m*_*x*_ to be smaller than *m*_*o*_.

Thus, (i) if *m_x_* < *m_o_* for the cells carrying reporter *x* under the appropriate stress *x*, then it is a TP; (ii) if *m_x_* > *m_o_* for the cells carrying reporter *x* under a stress other than *x*, then it is a TN; (iii) if *m_x_* < *m_o_* for the cells carrying reporter *x* under a stress other than *x*, then it is an FP; and (iv) if *m_x_* > *m_o_* for the cells carrying reporter *x* under a stress *x*, then it is an FN.

## Data Availability

A data package was deposited in Dryad with flow cytometry, microscopy, and spectrophotometry data (doi:10.5061/dryad.b2rbnzsm8). Raw RNA-seq data wasdata were published in (23) and can be accessed using the NCBI GEO accession code GSE178281. The library of GR reporters is available for distribution in Addgene.

## SUPPLEMENTAL MATERIAL

Supplemental material msystems.00065-24-s0001.pdf Supplemental figures and tables.

ASM does not own the copyrights to Supplemental Material that may be linked to, or accessed through, an article. The authors have granted ASM a non-exclusive, world-wide license to publish the Supplemental Material files. Please contact the corresponding author directly for reuse.
