# Supplementary material for: A library of reporters of the global regulators of gene expression in Escherichia coli
Source: mSystems. 2024 Apr 30;9(6):e00065-24. doi: 10.1128/msystems.00065-24 (PMC11237500; doi:10.1128/msystems.00065-24)
Supplement: Supplemental material — Supplemental figures and tables. [file msystems.00065-24-s0001.pdf]

Supplementary information for

A library of reporters of the global regulators of gene expression in *Escherichia coli*

Suchintak Dash[1], Rahul Jagadeesan[1], Ines S.C. Baptista[1], Vatsala Chauhan[1], Vinodh Kandavalli[2], Samuel M.D. Oliveira[3] and Andre S. Ribeiro[1,*]

[1] Faculty of Medicine and Health Technology, Tampere University, 33520, Finland

[2] Department of Cell and Molecular Biology, Uppsala University, Uppsala, Sweden

[3] Joint School of Nanoscience and Nanoengineering, North Carolina A&T State University, Greensboro, 27401-NC, USA

* To whom correspondence should be addressed. E-mail: andre.sanchesribeiro@tuni.fi

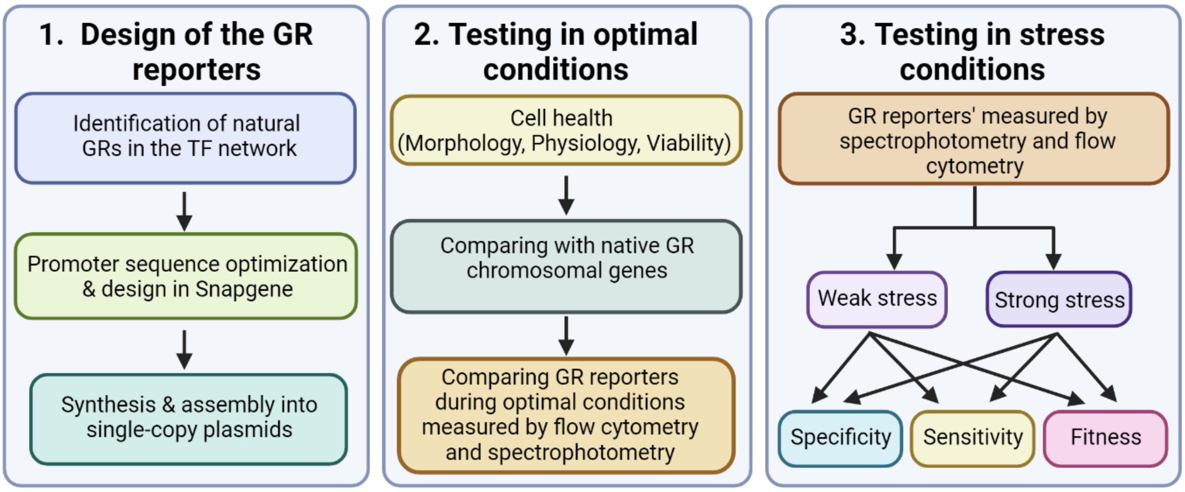

Figure S1: Graphical representation of the workflow.

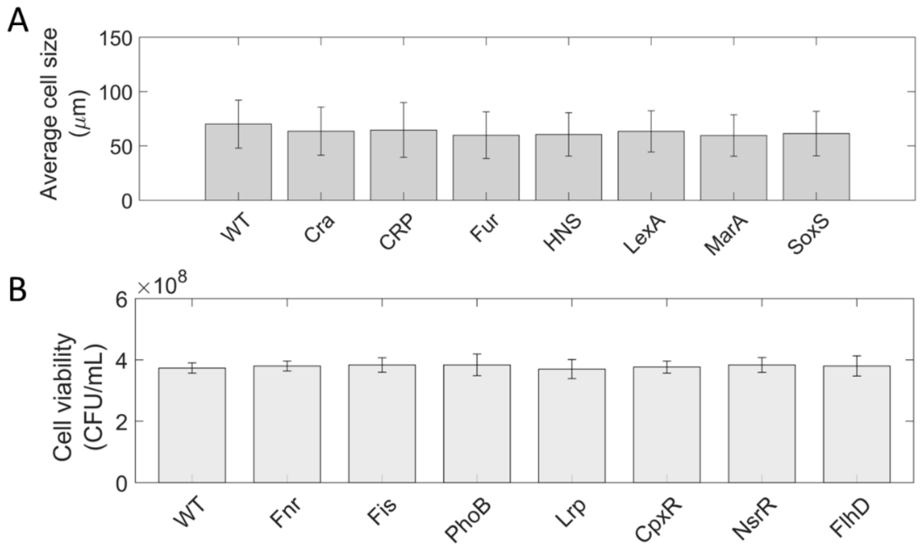

Figure S2: (A) Average cell size of several of the strains carrying GR plasmids and the WT (MG1655) strain, as measured by microscopy and image analysis. The error bars are the standard deviation (STDEV). **(B)** Viable cell count (as measured by CFU/mL) of the same strains. Error bars are the standard error of the mean (SEM) of 3 biological replicates.

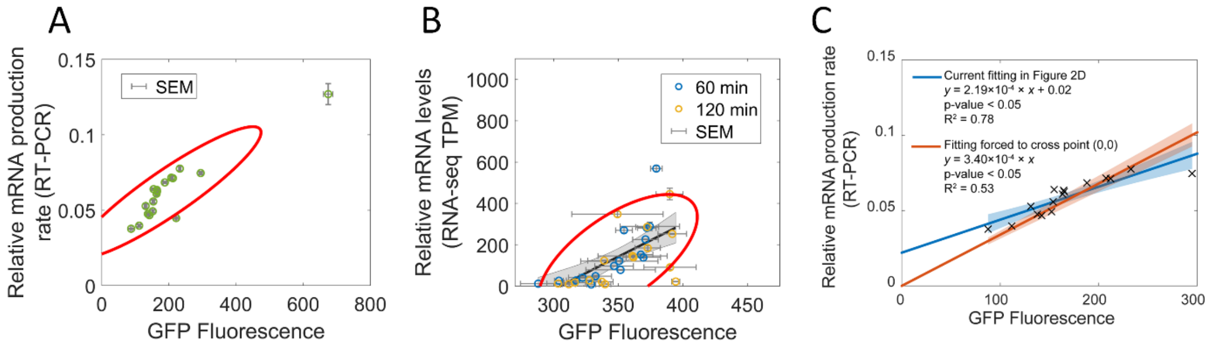

Figure S3: (A) Relative mRNA production of the GR reports (as measured by 1/ΔCq values from RT-PCR) plotted against GFP fluorescence measured by spectrophotometry. **(B)** RNA-seq TPM values at 60 min and at 120 min after placing the cells in fresh LB medium plotted against GFP fluorescence measured by spectrophotometry. The red ellipse is the 90% confidence ellipse, which defines the region with 90% of all datapoints. Datapoints in

(A) and (B) that fall outside of the ellipse were classified as being outliers and, therefore, were not considered when establishing the linear fit in Figures 2A and 2B in the main manuscript, respectively. **(C)** Scatter plot between relative mRNA production rates of the 16 native GR chromosomal genes and the GFP levels of the corresponding reporter plasmids (listed in Supplementary Table S1). We added two linear fits. The blue line is the standard linear fit (also shown in Figure 2A in the main manuscript). Meanwhile, the orange line was obtained by forcing the fitting to cross the origin of the plot. For both lines, we show their equation, along with the p-value and coefficient of determination, R^2 (Methods section “Fitting and statistical Analysis”). Moreover, the shadow areas of each line represent the 95% confidence intervals of the regression coefficients. These areas overlap for GFP levels ranging from 120 to 300, implying that the lines, in this region, are not statistically distinct.

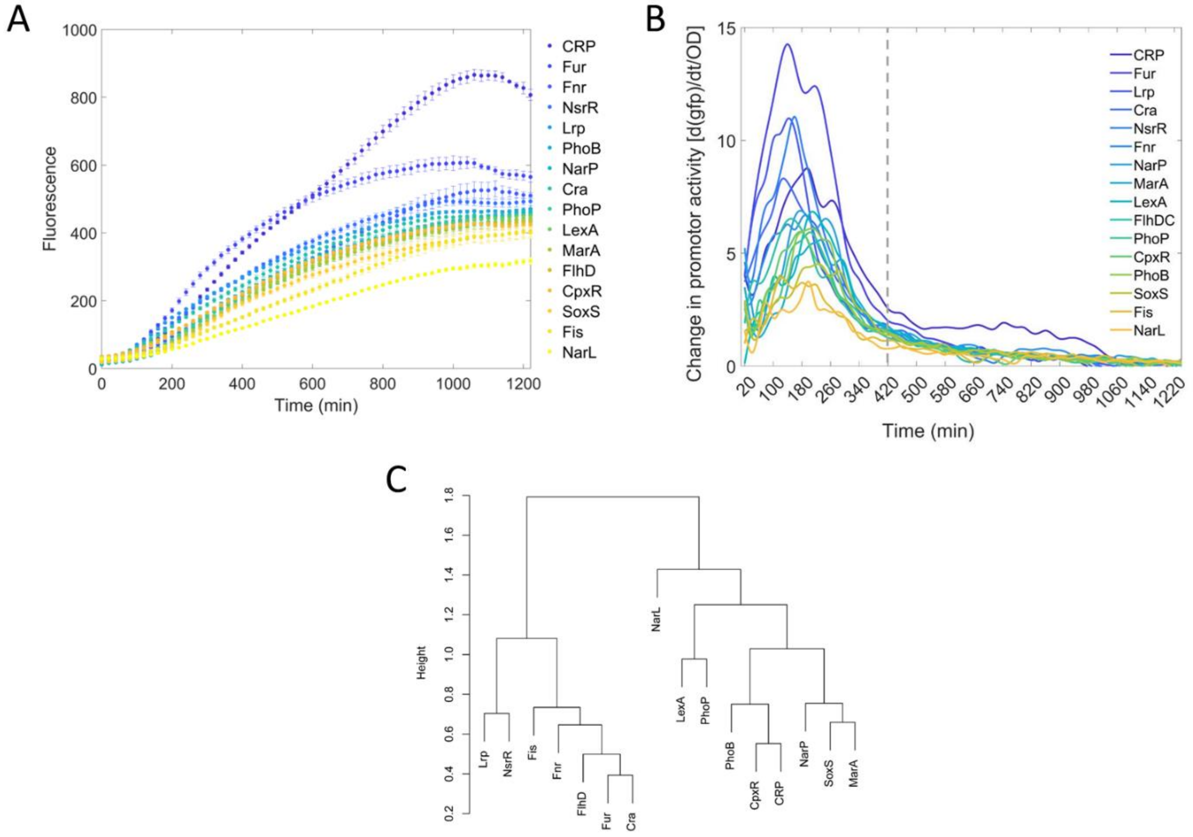

Figure S4: (A) Time lapse fluorescence of each of the 16 strains carrying the GR reporters, respectively. Data from when placing the overnight culture in the new medium until the cells reach the stationary growth phase. The average fluorescence of the WT cells over time (named ‘cellular background fluorescence’ in “Data Availability”) was subtracted from the data, but this had no significant influence on the results. **(B)** Change in GR promoter activity ($\frac{\frac{d(GFP)}{dt}}{OD}$) during standard growth conditions calculated from the fluorescence normalized by OD_{600} in Figure 3A. The dotted vertical line at 420 min marks the moment until when the data was used to obtain the heatmap in Figure 3B of the main manuscript. **(C)** Cluster dendrogram of the GR promoter activity over time, using the ‘pheatmap’ software R package, based on the empirical data until 420 min.

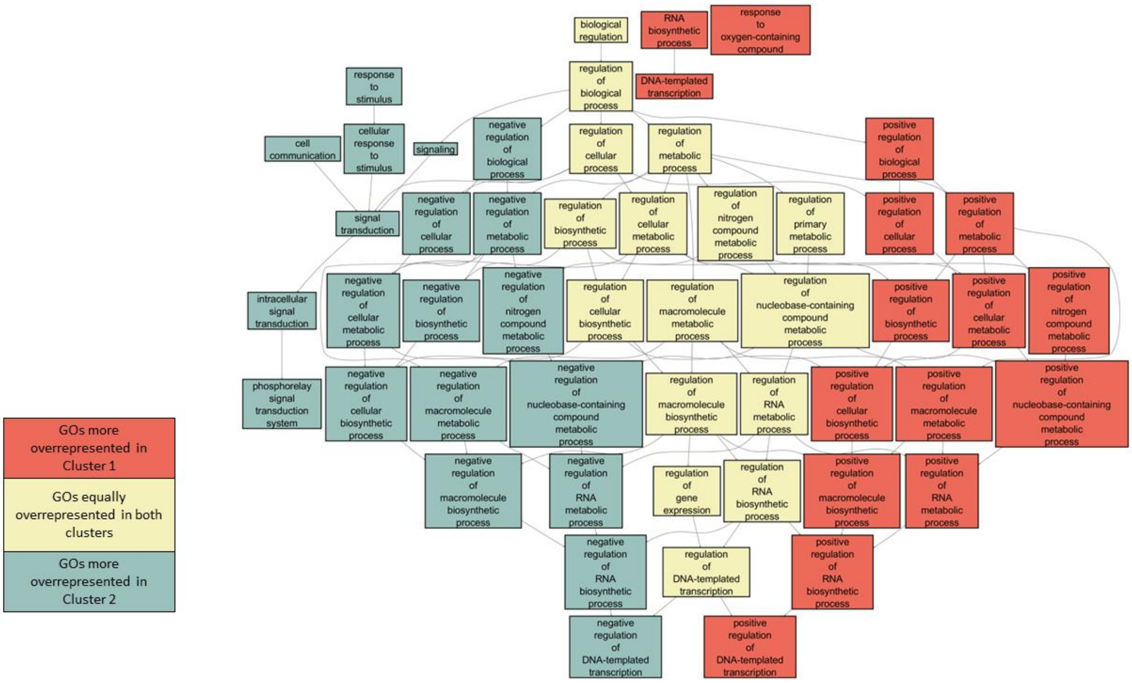

Figure S5: Overrepresented gene ontology (GO) terms and their ancestors for the genes controlled by the different GRs. The more general biological processes are connected to specific ontology terms by an arrow pointing to the latter. The ontologies that have the ancestors controlled by cluster 1 and/or 2 are colored differently.

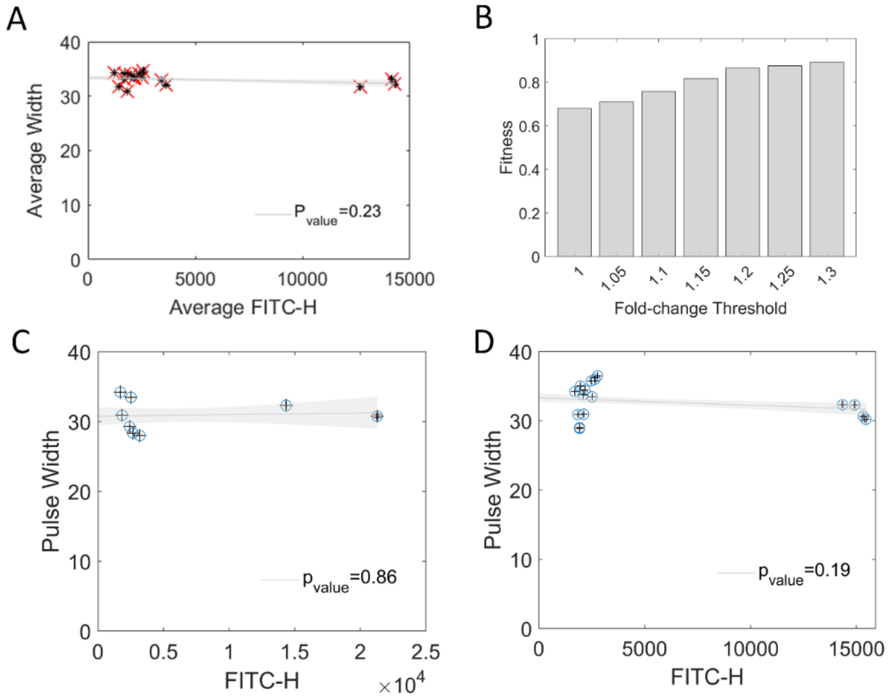

Figure S6: (A) Scatter plot of the average single-cell fluorescence (measured by FITC-H) of each strain carrying a GR reporter (data from 16 strains), plotted against the average pulse width of the same cell population (50,000 cells per population) in standard growth conditions. Also shown are the best fitting line and the p-value from a statistical test of whether the line differs from a horizontal line, using 'fitlm' in MATLAB. For a p-value > 0.05, we cannot conclude that the line differs from the horizontal line. **(B)** Fitness of the GR probes. Fitness is defined as the average between the specificity and the sensitivity values, as a function of a minimum threshold for fold changes, above which the probe is classified as being responsive to the stress. **(C-D)** Scatter plots of the average

cell pulse width plotted against the average cell fluorescence (measured by FITC-H) of each strain (Fur, MarA, LexA, and SosX) when **(C)** a given strain is subjected to the specific strong stress that it should respond to (Iron, Tetracycline, Kanamycin, and Oxidative stress, respectively), and, **(D)** when a given strain is subjected to the other three strong stresses that it should not respond to. Each data point is an average from 50,000 cells. Also shown are the best fitting lines and the p-value from a statistical test of whether the linear fit differs from a horizontal line, using 'fitlm' in MATLAB. For a p-value > 0.05, we cannot conclude that the line differs from the horizontal line.

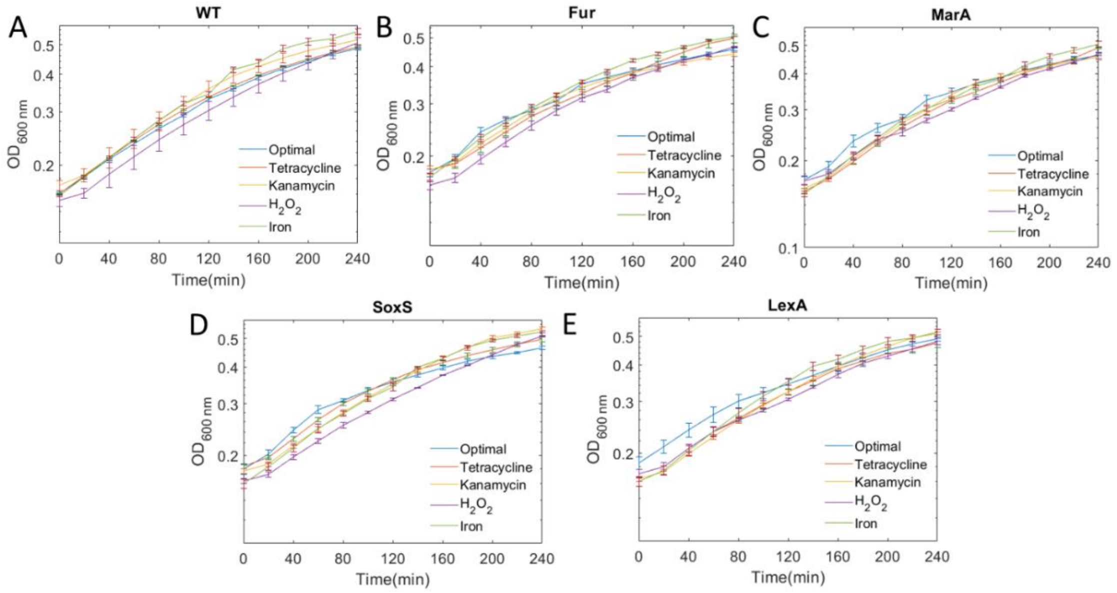

Figure S7: Growth curves of the **(A)** WT strain MG1655 and of cells containing GR reporters of **(B)** Fur, **(C)** MarA, **(D)** SoxS, and **(E)** LexA when subject to an external stress once reaching OD_{600} of 0.3 (from "0 min" onwards).

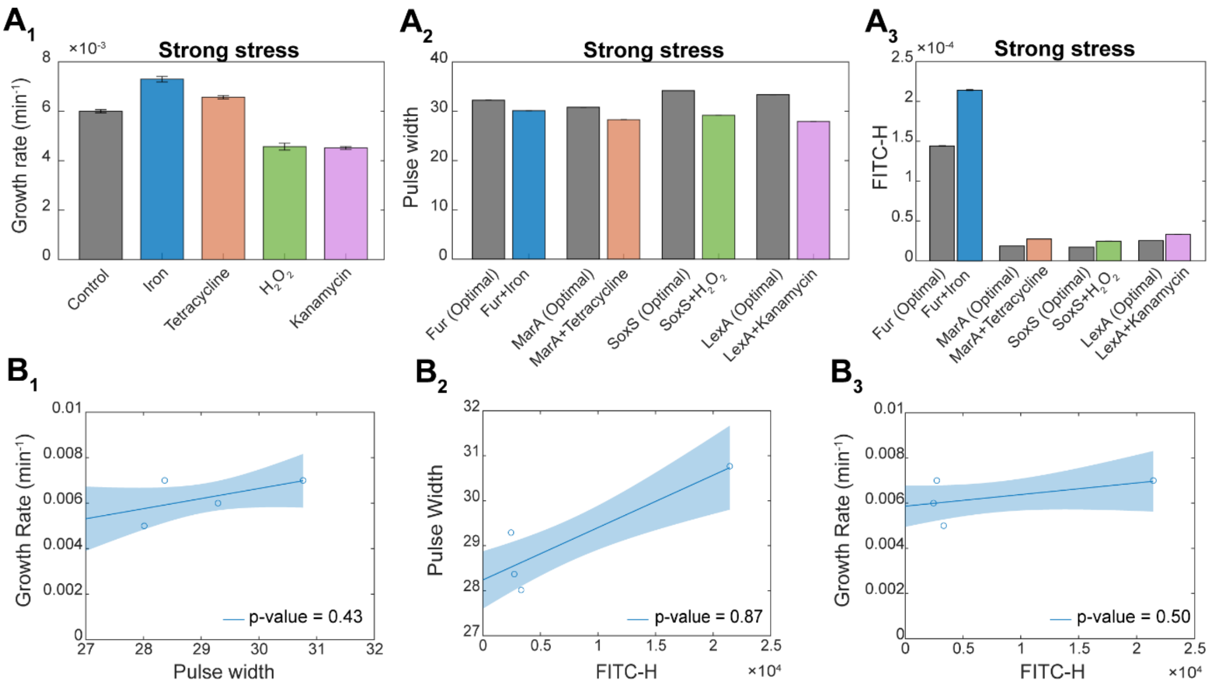

Figure S8: **(A_1)** Cell growth rates under strong stresses (Methods Section "Bacterial strains and stress conditions"). **(A_2)** Average pulse width (proxy for cell size). **(A_3)** Average FITC-H (single-cell fluorescence) of the strains carrying the GR reporters expectedly responsive to the stresses. Also shown are the scatter plots of **(B_1)** average growth rate versus average pulse width, **(B_2)** average FITC-H vs. average pulse width, and **(B_3)** average

growth rate vs. average FITC-H of the strains carrying the GR reporters responsive to the respective strong stresses.

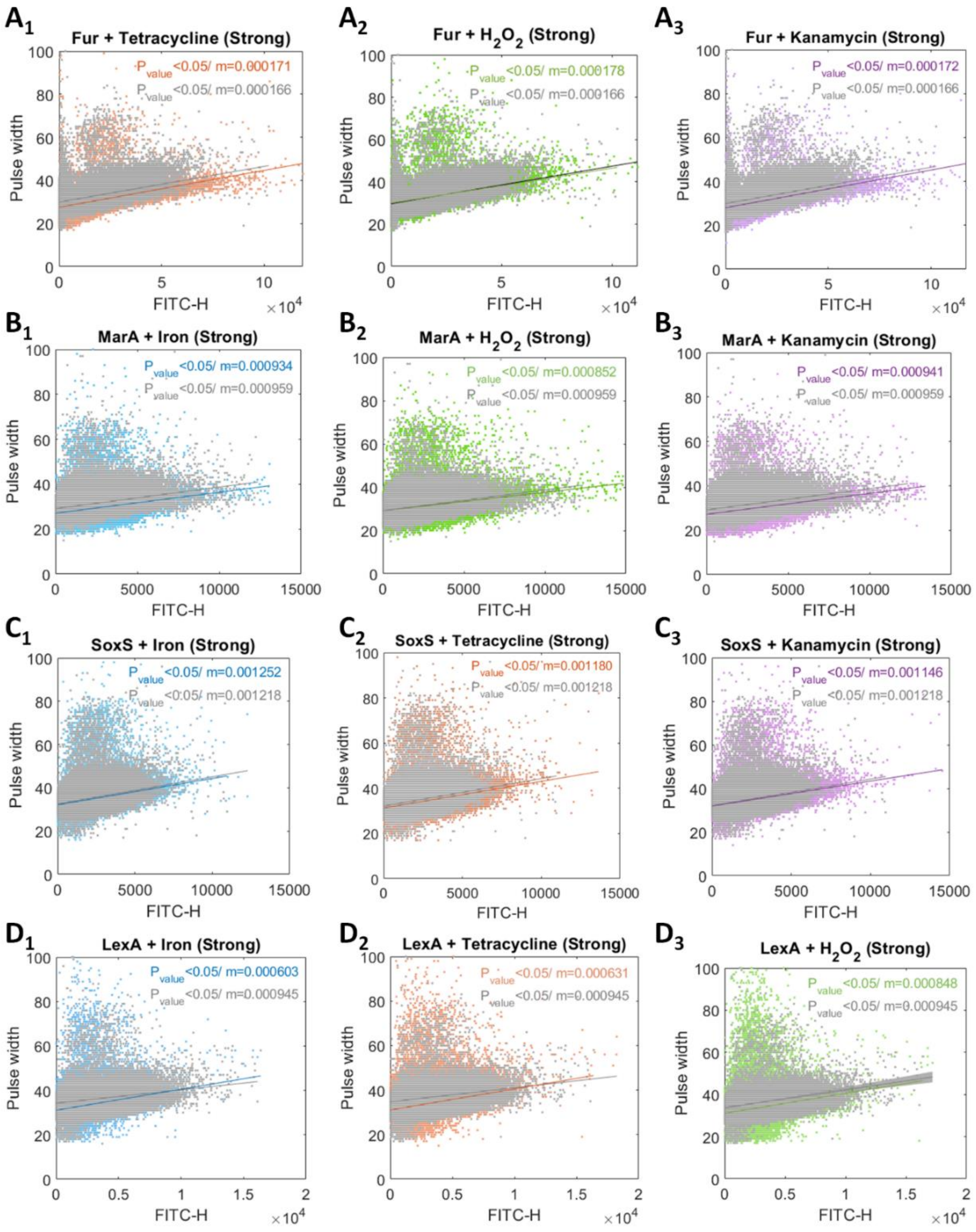

Figure S9: Single-cell GR reporter levels under strong stresses. Shown are single-cell expression levels (FITC-H) plotted against the corresponding single-cell pulse widths, measured by flow cytometry. Each figure shows data from cells subject to the stress (colored dots) and from cells not subject to the stress (grey dots). Also shown are the best linear fits to both cell cohorts. The p-values and slopes of the best fitting lines of each cohort are obtained from statistical tests determining whether the linear fits differ from horizontal lines, using the 'fitlm' function in MATLAB. For a p-value > 0.05, we cannot conclude that the line differs from a horizontal line. To compare whether the two slopes of the model are significantly different, a t-test statistic was calculated by

$\frac{m_1 - m_2}{\sqrt{SE_1^{\ 2} + SE_2^{\ 2}}}$, where m_1 and m_2 are the slopes of the fits to each cohort, respectively, while SE_1 and SE_2 are the corresponding standard errors, respectively. Finally, to assess if the result is not expected by random chance, a two-tailed *p*-value was calculated. Since the *p*-value was smaller than 0.05 in all cases, we concluded that the two slopes are not from the same distribution.

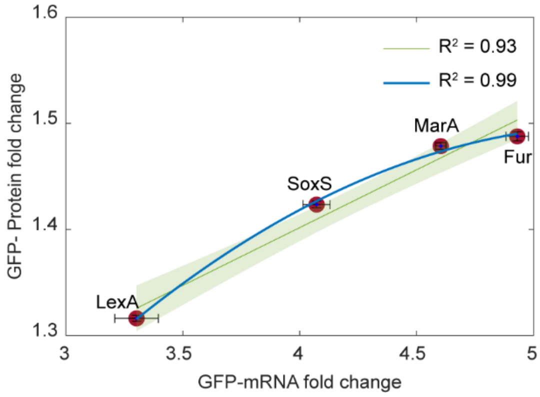

Figure S10: Scatter plot of the changes in GFP-Protein levels (measured by flow-cytometry) plotted against the changes in the corresponding GFP-mRNA levels (measured by RT-PCR). Each dot corresponds to a GR response strength to the stress it is known to be responsive to (MarA for tetracycline, LexA for Kanamycin, SoxS for H_2O_2, and Fur for iron stress). The fold changes are relative to the standard growth condition. Error bars are the standard errors of the mean. Also shown are the coefficients of determination, R^2, for a best fitting a green line and for a best fitting blue polynomial of order 2, respectively. The green shadow is the 95% confidence interval of the linear fitting.

Table S1: List of known global regulators of gene expression in *Escherichia coli* (genes coding for TFs that control 40 or more genes). Also shown are the number of genes that each GR controls (information from RegulonDB as of 19.04.2023) (1). It follows information on which libraries of synthetic constructs are these GR present in and whether the GR is subject to autoregulation.

Serial Number	GR names	No of genes directly regulated	No of genes indirectly regulated	GR reporter Library	TR Library (2)	YFP Fusion Library (3)	Autoregulation in GRs (1)
1	CRP	606	2546	Yes	yes		+,-
2	FNR	319	1139	Yes			-
3	IHF	261	695				None
4	Fis	245	984	Yes			-
5	HNS	201	441				None
6	ArcA	189	437				None
7	NarL	132	39	Yes			None
8	Fur	138	578	Yes	yes	Yes	-
9	Lrp	110	141	Yes			-
10	NsrR	86	169	Yes		Yes	None
11	Cra	82	748	Yes			None
12	FlhD	80	21	Yes		Yes	None
13	CpxR	71	162	Yes		Yes	+
14	NarP	66	0	Yes			None
15	PhoB	65	242	Yes	yes		+
16	LexA	61	66	Yes	yes		-
17	PhoP	59	237	Yes	yes		+,-
18	NtrC	56	30				None
19	MarA	52	89	Yes			+
20	ModE	46	132				None
21	SoxS	45	270	Yes	yes		-
22	PdhR	41	58		yes		-

Table S2: Biological processes that the GRs are known to affect.

Serial Number	GR	Biological Function
1	CRP	Glucose starvation (4)
2	FNR	Transition from aerobic to anaerobic metabolism (5)
3	Fis	DNA-binding protein involved in nucleoid organization (6)
4	HNS	Chromosome compaction (7)
5	NarL	Anaerobic respiration (8)
6	Fur	Global transcription repression as a response to iron excess (9)
7	Lrp	Adaptation to changes in the nutritional environment (10)
8	NsrR	Nitric oxide-sensitive repressor of transcription (11)
9	Cra	Carbon metabolism in nutrient limited environments (12)
10	FlhD	Flagellum biosynthesis (13)
11	CpxR	Biofilm formation (14)
12	NarP	Anaerobic respiration using nitrate (15)
13	PhoB	Inorganic phosphate metabolism regulation (16)
14	LexA	SOS response pathway regulation (17)

15	PhoP	Low Mg^{2+} concentration (18)
16	MarA	Antibiotic resistance (19)
17	PdhR	Pyruvate catabolism regulation (20)
18	SoxS	Superoxide stress protection (21)

Table S3: The table shows the outcome on the linear fitting of removing one data point (gene). Shown are the p-values of *t*-tests of the linear fits. For *p*-values < 0.05, we rejected the null hypothesis. Also shown are the R^2's of the goodness of fit of the line of the remaining 15 data points, after removing the datapoint.

Gene excluded from the linear fitting	*p*-value	R^2
CRP	< 0.05	0.75
CpxR	< 0.05	0.79
Cra	< 0.05	0.79
Fis	< 0.05	0.76
FlhD	< 0.05	0.79
Fnr	< 0.05	0.81
Fur	< 0.05	0.89
LexA	< 0.05	0.78
Lrp	< 0.05	0.77
MarA	< 0.05	0.79
NarL	< 0.05	0.73
NarP	< 0.05	0.79
NsrR	< 0.05	0.77
PhoB	< 0.05	0.79
PhoP	< 0.05	0.78
SoxS	< 0.05	0.78
None	< 0.05	0.78

Table S4: Euclidean distances between the expression level over time of all pairs of GRs. The distances are scaled by colors from red to white, corresponding to high to low.

	CpxR	SoxS	Fur	Lrp	NsrR	Cra	FlhD	CRP	Fnr	Fis	NarL	NarP	PhoB	LexA	PhoP	MarA
CpxR	0	0.95	0.69	1.15	0.70	0.68	0.73	0.55	0.66	0.78	1.01	0.90	0.70	1.10	1.00	1.01
SoxS		0	1.24	1.79	1.39	1.30	1.39	0.65	1.03	1.15	1.38	0.75	1.03	0.99	1.10	0.66
Fur			0	0.83	0.70	0.39	0.50	0.81	0.48	0.66	1.29	1.11	0.99	1.11	1.12	1.29
Lrp				0	0.70	0.65	0.74	1.35	0.95	1.08	1.43	1.60	1.54	1.58	1.47	1.71
NsrR					0	0.59	0.78	0.98	0.81	0.97	0.97	1.22	1.07	1.28	1.06	1.26
Cra						0	0.48	0.87	0.56	0.67	1.21	1.23	1.09	1.26	1.19	1.34
FlhD							0	0.86	0.65	0.62	1.34	1.17	1.03	1.20	1.28	1.37

CRP								0	0.62	0.84	1.11	0.71	0.75	0.89	0.95	0.81
Fnr									0	0.73	1.26	0.97	1.08	1.03	0.97	1.17
Fis										0	1.44	1.15	0.93	0.96	1.22	1.24
NarL											0	1.43	1.14	1.33	1.37	1.21
NarP												0	0.97	1.06	1.03	0.71
PhoB													0	0.96	1.25	1.01
LexA														0	0.98	0.87
PhoP															0	0.96
MarA																0

Table S5: Sensitivity and specificity of the GR reporters to weak stresses for different threshold values for LFCs.

Threshold LFC = 1						
**Stress**	**Sensitivity (TPR)**	**Specificity (TNR)**	**TP**	**FP**	**TN**	**FN**
Tetracycline	0.26	1.00	13	37	2	0
Kanamycin	0.35	1.00	13	24	15	0
Oxidative	0.34	1.00	13	25	14	0
Iron	0.52	0.97	12	11	28	1

Threshold LFC = 1.05						
**Stress**	**Sensitivity (TPR)**	**Specificity (TNR)**	**TP**	**FP**	**TN**	**FN**
Tetracycline	0.28	1.00	13	33	6	0
Kanamycin	0.41	1.00	13	19	20	0
Oxidative	0.41	1.00	13	19	20	0
Iron	0.65	0.94	11	6	33	2

Threshold LFC = 1.10						
**Stress**	**Sensitivity (TPR)**	**Specificity (TNR)**	**TP**	**FP**	**TN**	**FN**
Tetracycline	0.43	1.00	13	17	22	0
Kanamycin	0.50	1.00	13	13	26	0
Oxidative	0.52	0.97	12	11	28	1
Iron	0.71	0.92	10	4	35	3

Threshold LFC = 1.15						
**Stress**	**Sensitivity (TPR)**	**Specificity (TNR)**	**TP**	**FP**	**TN**	**FN**
Tetracycline	0.65	0.94	11	6	33	2
Kanamycin	0.68	1.00	13	6	33	0

Oxidative	0.67	0.92	10	5	34	3
Iron	0.80	0.88	8	2	37	5

Threshold LFC = 1.2						
**Stress**	**Sensitivity (TPR)**	**Specificity (TNR)**	**TP**	**FP**	**TN**	**FN**
Tetracycline	0.83	0.93	10	2	37	3
Kanamycin	0.70	0.86	7	3	36	6
Oxidative	0.88	0.86	7	1	38	6
Iron	1.00	0.87	7	0	39	6

Threshold LFC = 1.25						
**Stress**	**Sensitivity (TPR)**	**Specificity (TNR)**	**TP**	**FP**	**TN**	**FN**
Tetracycline	0.88	0.86	7	1	38	6
Kanamycin	0.75	0.84	6	2	37	7
Oxidative	1.00	0.83	5	0	39	8
Iron	1.00	0.85	6	0	39	7

Threshold LFC = 1.3						
**Stress**	**Sensitivity (TPR)**	**Specificity (TNR)**	**TP**	**FP**	**TN**	**FN**
Tetracycline	1.00	0.76	1	0	39	12
Kanamycin	1.00	0.80	3	0	39	10
Oxidative	1.00	0.80	3	0	39	10
Iron	1.00	0.78	2	0	39	11

REFERENCES

1. Tierrafría VH, Rioualen C, Salgado H, Lara P, Gama-Castro S, Lally P, Gómez-Romero L, Peña-Loredo P, López-Almazo AG, Alarcón-Carranza G, Betancourt-Figueroa F, Alquicira-Hernández S, Polanco-Morelos JE, García-Sotelo J, Gaytan-Nuñez E, Méndez-Cruz C-F, Muñiz LJ, Bonavides-Martínez C, Moreno-Hagelsieb G, Galagan JE, Wade JT, Collado-Vides J. 2022. RegulonDB 11.0: Comprehensive high-throughput datasets on transcriptional regulation in Escherichia coli K-12. Microb Genom 8.

2. Zaslaver A, Bren A, Ronen M, Itzkovitz S, Kikoin I, Shavit S, Liebermeister W, Surette MG, Alon U. 2006. A comprehensive library of fluorescent transcriptional reporters for Escherichia coli. Nat Methods 3:623–628.

3. Taniguchi Y, Choi PJ, Li G-W, Chen H, Babu M, Hearn J, Emili A, Xie XS. 2010. Quantifying E. coli Proteome and Transcriptome with Single-Molecule Sensitivity in Single Cells. Science 329:533–538.

4. Grainger DC, Hurd D, Harrison M, Holdstock J, Busby SJW. 2005. Studies of the distribution of Escherichia coli cAMP-receptor protein and RNA polymerase along the E. coli chromosome. Proc Natl Acad Sci U S A 102:17693–17698.

5. Unden G, Schirawski J. 1997. The oxygen-responsive transcriptional regulator FNR of Escherichia coli: the search for signals and reactions. Mol Microbiol 25:205–210.

6. Kelly A, Goldberg MD, Carroll RK, Danino V, Hinton JCD, Dorman CJ. 2004. A global role for Fis in the transcriptional control of metabolism and type III secretion in Salmonella enterica serovar Typhimurium. Microbiology 150:2037–2053.

7. Ueguchi C, Mizuno T. 1993. The Escherichia coli nucleoid protein H-NS functions directly as a transcriptional repressor. EMBO J 12:1039–1046.

8. Cai S-S, Zhang L-Q, Zhang Q, Ye B-C, Zhou Y. 2022. Acetylation of NarL K188 and K192 is involved in regulating Escherichia coli anaerobic nitrate respiration. Appl Microbiol Biotechnol 106:7209–7221.

9. Chen Z, Lewis KA, Shultzaberger RK, Lyakhov IG, Zheng M, Doan B, Storz G, Schneider TD. 2007. Discovery of Fur binding site clusters in Escherichia coli by information theory models. Nucleic Acids Res 35:6762–6777.

10. Landgraf JR, Wu J, Calvo JM. 1996. Effects of nutrition and growth rate on Lrp levels in Escherichia coli. J Bacteriol 178:6930–6936.

11. Partridge JD, Bodenmiller DM, Humphrys MS, Spiro S. 2009. NsrR targets in the Escherichia coli genome: new insights into DNA sequence requirements for binding and a role for NsrR in the regulation of motility. Mol Microbiol 73:680–694.

12. Shimizu K. 2013. Regulation Systems of Bacteria such as Escherichia coli in Response to Nutrient Limitation and Environmental Stresses. Metabolites 4:1–35.

13. Gauger EJ, Leatham MP, Mercado-Lubo R, Laux DC, Conway T, Cohen PS. 2007. Role of motility and the flhDC Operon in Escherichia coli MG1655 colonization of the mouse intestine. Infect Immun 75:3315–3324.

14. Dudin O, Geiselmann J, Ogasawara H, Ishihama A, Lacour S. 2014. Repression of flagellar genes in exponential phase by CsgD and CpxR, two crucial modulators of Escherichia coli biofilm formation. J Bacteriol 196:707–715.

15. Brosse A, Boudry P, Walburger A, Magalon A, Guillier M. 2022. Synthesis of the NarP response regulator of nitrate respiration in Escherichia coli is regulated at multiple levels by Hfq and small RNAs. Nucleic Acids Res 50:6753–6768.

16. Marzan LW, Shimizu K. 2011. Metabolic regulation of Escherichia coli and its phoB and phoR genes knockout mutants under phosphate and nitrogen limitations as well as at acidic condition. Microb Cell Fact 10:39.

17. D'Ari R. 1985. The SOS system. Biochimie 67:343–347.

18. Groisman EA. 2001. The pleiotropic two-component regulatory system PhoP-PhoQ. J Bacteriol 183:1835–1842.

19. Sulavik MC, Gambino LF, Miller PF. 1995. The MarR repressor of the multiple antibiotic resistance (mar) operon in Escherichia coli: prototypic member of a family of bacterial regulatory proteins involved in sensing phenolic compounds. Mol Med 1:436–446.

20. Anzai T, Imamura S, Ishihama A, Shimada T. 2020. Expanded roles of pyruvate-sensing PdhR in transcription regulation of the Escherichia coli K-12 genome: fatty acid catabolism and cell motility. Microb Genom 6.

21. Li Z, Demple B. 1994. SoxS, an activator of superoxide stress genes in Escherichia coli. Purification and interaction with DNA. J Biol Chem 269:18371–18377.
